# Revisiting the PCR‐Based Molecular Approaches for Mycotoxigenic *Fusarium* Species Detection and Quantification in Wheat

**DOI:** 10.1111/1541-4337.70612

**Published:** 2026-08-28

**Authors:** Kavitha Vijeandran, Alexey Larionov, Carla Cervini, Katrina Campbell, Sean Walkowiak, Matias Pasquali, Florence Richard‐Forget, Neil Brown, Lindy Joy Rose, Carol Verheecke‐Vaessen

**Affiliations:** ^1^ Faculty of Engineering and Applied Sciences Cranfield University Cranfield UK; ^2^ Magan Centre of Applied Mycology Cranfield University Cranfield UK; ^3^ Institute for Global Food Security, School of Biological Sciences Queen's University Belfast Belfast UK; ^4^ Department of Genomic Medicine University of Cambridge Cambridge UK; ^5^ Grain Research Laboratory Canadian Grain Commission Winnipeg Manitoba Canada; ^6^ Department of Food, Environmental and Nutritional Sciences University of Milan Milan Italy; ^7^ INRAE, Mycology and Food Safety (MycSA) Villenave d'Ornon France; ^8^ Department of Life Sciences University of Bath Bath UK; ^9^ Department of Plant Pathology Stellenbosch University Stellenbosch South Africa

**Keywords:** applied mycology, bioinformatics, *Fusarium* genomic resources, fusarium head blight, molecular tests, polymerase chain reaction, trichothecene, zearalenone

## Abstract

*Fusarium* species cause yield losses in wheat production through fusarium head blight (FHB) and the associated contamination of regulated mycotoxins, such as trichothecenes and zearalenone. Despite the widespread use of PCR‐based molecular approaches for *Fusarium* detection, quantification, and chemotyping, most primers were developed prior to both modern phylogenetic reclassification and the availability of high‐quality genome assemblies, leaving their specificity and robustness largely untested. Existing PCR‐ and qPCR‐based assays for *Fusarium* detection in wheat were reviewed and re‐evaluated in silico using a curated genome panel. Of 53 species‐specific primer pairs, 14 (26.4%) achieved high‐specificity grades (A–B), whereas 25 (47.2%) were lower performing (D–E), mainly due to cross‐reactivity or inconsistent target amplification. Chemotype assays targeting TRI and ZEN genes showed stronger agreement with reported chemotypes, especially for informative TRI loci such as *Tri3*, *Tri7*, and *Tri12*. To support improved qPCR assay design and reporting, we propose FusaMIQE, a *Fusarium*‐adapted framework based on MIQE 2.0 guidelines, tailored to the specific challenges of *Fusarium* diagnostics in wheat. Together, this manuscript provides the first in silico assessment of PCR/qPCR primers as diagnostic tools for FHB pathogens and associated recommendations for good practice (FusaMIQE) of *Fusarium* diagnostics in wheat.

## 1 Introduction

Global wheat production represents an estimated 787 million tonnes in 2023/24 (Food and Agriculture Organization of the United Nations (FAO) 2024). Fusarium head blight (FHB) remains one of the most significant threats to wheat production, causing losses of 10%–70% in epidemic years (Khan et al. 2020). Beyond yield reduction, FHB is of particular concern because infection can reduce grain quality and marketability. Additionally, associated mycotoxins can limit suitability for food, feed, and processing, pose serious risks to human and animal health, and are strictly regulated in the European Union (EU) and globally (Johns et al. 2022). Unlike many fungal diseases caused by a single species, FHB represents a complex disease involving multiple *Fusarium* species, with up to 17 phylogenetically distinct species contributing to infection, each differing in pathogenicity and mycotoxin production potential (Alisaac and Mahlein 2023).

The genus *Fusarium* comprises a large and taxonomically complex group of filamentous fungi that includes plant pathogens, endophytes, saprophytes, and opportunistic animal pathogens and has been repeatedly redefined taxonomically as new data and concepts have emerged. Early classifications, based mainly on morphology, recognized hundreds to more than a thousand putative species but offered limited insights into evolutionary relationships (Summerell 2019). More recently, multilocus and genome‐scale phylogenetics have reshaped this picture, showing that the genus comprises over 400 species grouped into well‐supported species complexes (O'Donnell et al. 2022).

The development of polymerase chain reaction (PCR) has revolutionized methods for *Fusarium* detection. Many different types of PCR‐based molecular markers have been developed over the past 25 years. Early molecular assays frequently relied on random amplified polymorphic DNA (RAPD) markers, later converted into sequence‐characterized amplified region (SCAR) markers to improve reproducibility (Baturo‐Ciesniewska and Suchorzynska 2011). Notably, many early PCR‐based assays were developed with limited genomic resources and were designed to align with the morphology‐based classifications prevailing at the time. In most cases, PCR products were not sequenced, leaving assay specificity largely unvalidated at the sequence level. As a result, their specificity and robustness remain uncertain when evaluated against current genomic resources.

Despite these limitations, numerous *Fusarium*‐specific legacy primers reported decades ago are still in common use. For example, the Fg16F/Fg16R primer pair developed in 1998 (P. Nicholson et al. 1998) continues to be widely used to distinguish *F. graminearum* from other *Fusarium* species (Wang et al. 2023; Zuzak et al. 2018; Spanic et al. 2010). The subsequent development of quantitative PCR (qPCR) further extended these capabilities, enabling not only detection but also biomass estimation of *Fusarium* species in wheat samples. Yet these assays inherited many of the same design limitations as their conventional PCR predecessors.

In parallel, the rapid expansion of *Fusarium* genomic resources and improved understanding of species boundaries have revealed extensive intra‐ and interspecies diversity. This raises critical questions regarding the continued reliability of legacy molecular assays developed prior to modern phylogenetic frameworks. Therefore, this review aims to critically evaluate in silico the PCR‐based molecular approaches used for the detection, identification, and quantification of *Fusarium* species in wheat, in the context of contemporary *Fusarium* taxonomy and rapidly expanding genomic resources. Emphasis is placed on reassessing legacy primers developed prior to the modern phylogenetic reclassification, evaluating their specificity and robustness against the current genomic data.

The review initially describes the challenges in molecular characterization of *Fusarium* species, followed by the methodology describing the search strategy for the identification of published PCR‐based assays, current *Fusarium* genomic resources, and the algorithm that we applied to evaluate PCR assays against the selected genomes. Following this, the results of the published PCR assays assessment are presented. Finally, the review concludes with a discussion of the findings and recommendations for the future development of PCR‐based molecular assays for *Fusarium* analysis.

## 2 Challenges in *Fusarium* Molecular Analysis

Challenges in *Fusarium* molecular analysis stem from (i) the diversity of the mycotoxin‐producing pathways, (ii) the ever‐changing taxonomy of *Fusarium*, and (iii) the multiplicity of methods applied for the molecular analysis of *Fusarium*.

### 2.1 Mycotoxins in *Fusarium*: Types, Toxicology, and Regulations

In wheat, FHB represents not only a disease of spikes but also a contamination problem because infection by multiple *Fusarium* species (Alisaac and Mahlein 2023) typically results in complex mycotoxin mixtures in the harvested grains. These toxins drive most of the economic and regulatory impact of FHB, influencing grain usability, trade, and food and feed safety (Johns et al. 2022) decisions more directly than yield loss alone. Consequently, molecular detection systems for *Fusarium* in wheat must be evaluated not only for their taxonomic resolution but also for how well they capture the underlying mycotoxin risk associated with different species and chemotypes.

Among the most concerning mycotoxin groups are trichothecenes and zearalenone (ZEN), due to their prevalence in infected cereals and their toxicological effects. Trichothecenes represent the most important group in wheat and are divided into four major structural types, of which Types A and B are the most prevalent. Type A mycotoxins include T‐2 toxin, HT‐2 toxin, and diacetoxyscirpenol, while Type B includes deoxynivalenol (DON) and nivalenol (NIV) (Kimura et al. 2007). The less common Type C and Type D trichothecenes are typically produced by non‐*Fusarium* genera. In addition to trichothecenes, *Fusarium* species produce ZEN and its metabolites (α‐zearalenol and β‐zearalenol), which constitute a distinct class of resorcylic acid lactones with estrogenic activity (Ropejko and Twarużek 2021). Fumonisins, predominantly produced by *F. verticillioides* and *F. proliferatum*, and moniliformin represent other significant *Fusarium* mycotoxins, though their occurrence is predominantly in maize (Alexander et al. 2009).

The specific combination of mycotoxins produced by a *Fusarium* isolate is termed its chemotype, which is primarily determined by the presence and allelic variants of biosynthetic genes within the mycotoxin gene clusters or the production capacity of the isolates. Importantly, chemotype variation can occur among isolates within the same species. For example, within the *F. graminearum* species complex, isolates are classified into distinct chemotypes based on their trichothecene profiles: 3‐ADON (3‐acetyldeoxynivalenol), 15‐ADON (15‐acetyldeoxynivalenol), NIV, or NX (3α‐acetoxy‐7α,15‐dihydroxy‐12,13‐epoxytrichothec‐9‐ene) producers, each reflecting different allelic variants of genes such as *Tri1*, *Tri7*, and *Tri13* within the trichothecene biosynthetic gene cluster (BGC) (Alexander et al. 2011). Chemotype distribution varies remarkably across geographic regions and is influenced by host crop differences, environmental conditions, and evolutionary history of local *Fusarium* populations, with important implications for mycotoxin risk assessment and detection strategies (Varga et al. 2015).

#### 2.1.1 Trichothecenes: Structure, Occurrence, and Biosynthesis

Trichothecenes are characterized by a conserved tetracyclic 12,13‐epoxytrichothec‐9‐ene (EPT) core structure, with the C‐12,13 epoxide group being essential for their biological activity and toxicity, where Type A trichothecenes lack a carbonyl group at C‐8, whereas Type B trichothecenes possess a carbonyl or hydroxyl group at this position (McCormick et al. 2011). In wheat, trichothecenes are primarily produced by *F. graminearum* sensu stricto, *F. culmorum*, *F. asiaticum*, *F. sporotrichioides*, *F. langsethiae*, and *F. poae*, with species prevalence varying across geographic regions (Alisaac and Mahlein 2023) and cereal contamination.

Historically, Type A trichothecenes such as T‐2 and HT‐2 have been reported in wheat at a lower incidence than DON and ZEN (Pettersson 1995). However, analysis of European monitoring data showed that since ∼2010, the relative proportion of T‐2 and HT‐2 in contaminated wheat has risen, suggesting a shift in *Fusarium* populations, likely due to increased co‐infection by *F. sporotrichioides* (Johns et al. 2022). A survey of Italian durum wheat from 2013 to 2015 reported T‐2 and HT‐2 in up to 83% of samples (mean ∼310 µg/kg), with 60% of Central Italy and 100% of Southern Italy samples contaminated in 2013–2014 (Haidukowski et al. 2022). Of the Type B trichothecenes, DON—also known as vomitoxin—is the most prevalent trichothecene globally, commonly detected in wheat samples in surveillance studies, and often co‐occurring with its acetylated derivatives, 3‐ADON and 15‐ADON, that become deacetylated in planta (Schmeitzl et al. 2015; Gruber‐Dorninger et al. 2019). In contrast, NIV is less common in most wheat‐growing regions. This pattern primarily reflects regional differences in *Fusarium* species composition and chemotype structure. Regions where *F. asiaticum*, with most strains of this species being of the NIV chemotype, is the dominant FHB pathogen, like parts of China, Japan, and Korea, consistently show higher NIV levels (Lee et al. 2020).

Besides parent compounds, trichothecenes also occur as conjugated or “masked” mycotoxins, formed through plant detoxification processes in which the toxin can be conjugated with polar moieties such as glucose (Berthiller et al. 2013). Berthiller et al. (2005) provided the first definitive evidence for the natural occurrence of deoxynivalenol‐3‐glucoside (DON‐3‐G) in *Fusarium*‐infected wheat and maize. These masked forms escape routine analytical detection (e.g., ELISA) due to their increased polarity and lack of commercially available standards. They, however, can pose an additional threat to food safety as they could be hydrolyzed back to their toxic parent compounds during digestion. In naturally contaminated wheat samples, DON‐3‐G was detected at levels corresponding to approximately 4%–12% of free DON, exceeding concentrations of acetylated DON derivatives in some batches (Berthiller et al. 2005).

Trichothecenes are synthesized via a biosynthetic pathway encoded by the BGCs comprising approximately 15 genes typically distributed across three chromosomal loci (e.g., chromosomes 1, 2, and 3 in *F. graminearum*) (Khaneghah et al. 2018). The key genes include *Tri5*, encoding trichodiene synthase, and multiple cytochrome P450 monooxygenases such as *Tri1*, *Tri4*, and *Tri11*, which catalyze successive oxygenation steps. The distinction between Type A and Type B trichothecenes is largely governed by allelic variation in *Tri1*, which determines hydroxylation at C‐7 and C‐8. Type B producers carry a *Tri1* allele (*FgTri1*) capable of hydroxylating both positions, whereas Type A producers typically have *FsTri1*, which catalyzes hydroxylation at the C‐8 position only (McCormick et al. 2011). Allelic variation in the *Tri1* gene has been demonstrated to result in the production of NX toxin (Varga et al. 2015). The *Tri8* and *Tri16* genes encode acetyltransferases; variation within these genes contributes to chemotype variation, where alternate alleles of *Tri8* result in 15‐ADON or 3‐ADON, and *Tri16* function results in T‐2 toxin (Peplow et al. 2003). *Tri6* and *Tri10* encode transcription factors that regulate the expression of other genes in the trichothecene BGC, while *Tri12* encodes a transporter for toxin export (Seong et al. 2009). Due to the extensive genetic polymorphism observed within the trichothecene BGC, particularly in genes such as *Tri3*, *Tri5*, *Tri7*, *Tri8*, *Tri12*, *Tri13*, and *Tri16*, numerous molecular genotyping assays have been developed to detect and quantify *Fusarium* species toxin biosynthesis potential.

#### 2.1.2 ZEN: Structure, Occurrence, and Biosynthesis

ZEN is a nonsteroidal, estrogenic mycotoxin belonging to the class of resorcylic acid lactones, characterized by a macrolactone ring with phenolic hydroxyl groups that enable binding to estrogen receptors (Hueza et al. 2014). Due to its estrogen‐mimicking characteristics, ZEN affects endocrine functions and is associated with reproductive disorders in humans and animals. ZEN is chemically stable, lipophilic, and heat resistant, allowing it to persist through food processing and storage. In wheat, ZEN is mainly produced by *F. graminearum* and *F. culmorum* (Ropejko and Twarużek 2021). ZEN is a common *Fusarium* contaminant in cereal, but its prevalence is highly variable according to the country, year, and commodity fraction. Across Europe, as documented by the European Food Safety Agency food‐chain monitoring data (2010–2019), ZEN remains a frequent co‐contaminant along with DON in both food and feed wheat, consistent with their joint production by key FHB species such as *F. graminearum* and *F. culmorum* (Johns et al. 2022). The EU‐wide Scientific Co‐operation Task 3.2.10 data submitted by Member States detected ZEN in 32% of all analyzed food samples overall, and within cereal categories, it was reported in approximately ∼30% of wheat samples, 24% of wheat milling fraction, and ∼11% of wheat‐based products (Gareis et al. 2023).

Beyond its free form, ZEN exists in multiple biologically active forms, including its phase I metabolites α‐zearalenol and β‐zearalenol, with α‐zearalenol being significantly more potent than ZEN itself (Ropejko and Twarużek 2021). Similar to trichothecenes, during plant metabolism and food processing, ZEN can also be transformed into “masked” derivatives such as ZEN‐14‐glucoside, which escape detection by standard assays but can be converted back into toxic forms in the digestive tract (Liu and Applegate 2020). As the presence of masked mycotoxins represents a significant analytical blind spot for their direct biochemical detection, molecular approaches targeting ZEN biosynthetic genes provide a complementary strategy to assess the toxigenic potential of *Fusarium* species. In *Fusarium*, the ZEN BGC spans ∼50 kb and includes four essential genes: two genes encoding polyketide synthesis enzymes (PKS13 and PKS4) and two accessory genes (ZEB1, ZEB2), which encode regulatory proteins (Nahle et al. 2021). Thus, the ZEB2‐mediated feedback ensures self‐limiting toxin production once a threshold is reached. Other environmental factors and regulatory mechanisms, such as the velvet complex and cAMP‐PKA signaling, are also involved in ZEN BGC regulation as reviewed by Viñas and Karlovsky (2025).

#### 2.1.3 Regulatory Framework

*Fusarium* mycotoxins are associated with distinct and well‐characterized adverse health effects. DON can induce vomiting and immunosuppression in both humans and livestock (Sobrova et al. 2010). NIV is considered more cytotoxic than DON and can impair intestinal barrier integrity, immune function, and hematopoiesis, while T‐2 and HT‐2 toxins exhibit particularly high acute toxicity, affecting the immune system and multiple organ systems in humans (Meneely et al. 2023). ZEN acts as an endocrine disruptor by mimicking estrogen and affecting hormonal balance (Kowalska et al. 2016). The EU currently applies some of the most stringent limits, with maximum levels in unprocessed wheat set at 1250 µg/kg for DON, 100 µg/kg for ZEN, and 1000 µg/kg (total) for T‐2 and HT‐2 toxins (European Commission 2023). In contrast, Codex Alimentarius Commissions (CAC) guidelines are generally less restrictive, permitting up to 0.2 mg/kg for DON in raw cereals (Chen et al. 2021) destined for further processing, and have not established consistently harmonized maximum levels for ZEN across cereal commodities, while no CAC maximum limits currently exist for T‐2 and HT‐2 toxins. Notably, despite NIV being more cytotoxic than DON, it currently lacks an established EU and CAC regulatory maximum limit.

### 2.2 Evolution of *Fusarium* Taxonomy and Detection Methods

The genus *Fusarium*, established by Link in 1809, historically encompassed over 1000 species based solely on morphological variation. While dual naming conventions of *Gibberella* (teleomorph, sexual) and *Fusarium* (anamorph, asexual) exist in the literature based on the sexual stages of fungi, this has largely been abandoned in favor of the genus name *Fusarium*, since most species within the genera remain in their asexual forms (Geiser et al. 2013). Early taxonomists like Wollenweber and Reinking (1935) attempted to bring order by organizing *Fusarium* into 16 sections and 65 species (Fourie et al. 2011). In contrast, the “nine‐species” concept proposed by Snyder and Hansen (1945) in the 1940s lumped the various forms of *Fusarium* into just nine broad species (Summerell 2019). Taxonomic refinement accelerated in the late 20th century with the advent of biological and phylogenetic species concepts. Mating studies in the 1970s to 1990s revealed cryptic biological species within morphological species, leading to a more nuanced classification (Summerell and Leslie 2011).

The implementation of the “One Fungus, One Name” principle, which mandates a single accepted name for each fungal species regardless of its life stage, led to more taxonomy revisions in the year 2013 (Geiser et al. 2013). However, the most profound reshaping of *Fusarium* taxonomy has come from the recent advances in molecular phylogenetics, suggesting that the *Fusarium* genus comprises over 400 distinct species organized into 23 monophyletic species complexes, which can be hierarchically nested within one another (O'Donnell et al. 2022). For instance, the *F. graminearum* species complex (the primary cause of FHB worldwide) is nested within the *F. sambucinum* species complex; the species prevalence within the species complexes varies between different world regions and is constantly evolving (Laraba et al. 2021).

#### 2.2.1 Early Detection Methods: Morphology and Culture‐Based

Historically, *Fusarium* detection was based on morphological characteristics following pathogen isolation. However, this approach is time‐consuming and requires highly specialized taxonomic expertise (Karelov et al. 2021). Multiple official guidelines and protocols still incorporate morphology‐based detection for *Fusarium* in wheat (Leslie and Summerell 2006). In the United Kingdom, the Agriculture and Horticulture Development Board (AHDB) (2025) recommends an agar plate assay to detect seedborne *Fusarium*. It could be noted that detection based solely on morphology can lead to underrepresentation or complete masking of less dominant species, including *F. langsethiae*, complicating assessment of the diversity. However, despite these limitations, many current approaches still include morphology to complement molecular PCR‐based techniques for *Fusarium* identification (A. H. M. Kumar et al. 2024). Other approaches such as matrix‐assisted laser desorption/ionization time‐of‐flight mass spectrometry (MALDI‐TOF MS) have been explored as a rapid method for fungal identification (Laraba et al. 2021). While MALDI‐TOF can enable rapid genus‐level identification of *Fusarium*, its discriminatory power remains limited within species complexes.

Although several approaches are available for the detection and characterization of *Fusarium* contamination in wheat, including morphology‐based identification, culture‐dependent isolation, immunological assays, chromatographic mycotoxin analysis, and sequencing‐based methods, a comprehensive comparison of all diagnostic platforms is beyond the scope of this review.

#### 2.2.2 Internal Transcribed Spacer (ITS) Barcoding and Multilocus Sequencing

Sequencing of the ITS region of ribosomal DNA was suggested as the universal fungal barcode by Schoch et al. (2012), due to the high interspecies variability and ease of amplification for this region. While ITS sequencing remains widely used for fungal identification in general, it lacks the resolution to distinguish *Fusarium* at the species level. The multiple copies of the ribosomal operon per genome, intragenomic polymorphism of the ITS region, and horizontal gene transfer further complicate its use.

To address these shortcomings, *Fusarium* researchers shifted toward multilocus DNA barcoding. Multi‐Locus Sequence Typing (MLST) offers improved resolution through analysis of five to seven housekeeping genes (Desoubeaux et al. 2018). Building on this, the Genealogical Concordance Phylogenetic Species Recognition (GCPSR) framework emerged as the current gold standard for *Fusarium* species delimitation, utilizing over 10 unlinked loci to resolve cryptic species via genealogical concordance (Aoki et al. 2012). O'Donnell et al. (2015) suggested a multigene amplicon sequencing panel for the *Fusarium* genus analysis, including protein‐coding regions of the TEF1 (translation elongation factor 1‐α), RPB1, and RPB2 genes, which are single copy and informative at or near the species level. This gene panel has become a standard in GCPSR‐based studies and is recommended over ITS for reliable, sequence‐based diagnostics. To support this, the Fusarium‐ID database (https://www.fusarium.org/) was developed as a curated repository of the relevant *Fusarium* gene sequences and reference strain data, offering a curated platform for species‐level identification of *Fusarium* (Geiser et al. 2004).

#### 2.2.3 Functional Detection and Mycotoxin Biosynthesis Genes

As genomic data expanded, attention shifted from taxonomic identification toward functional detection, particularly targeting mycotoxin BGCs to predict toxigenic potential. Early examples include PCR assays targeting trichothecene biosynthetic genes such as *Tri5*, *Tri6*, and *Tri13* to infer toxigenic potential and chemotype, rather than the species identity (P. C. Nicholson et al. 2004; Chandler et al. 2003; Jennings et al. 2004). This functional approach allowed for rapid screening of wheat and grain samples for mycotoxin risk without the need for full species resolution and remains widely applied in surveillance and risk assessment programs.

#### 2.2.4 Advances in PCR‐Based Molecular Detection of *Fusarium*

**FIGURE 1:**
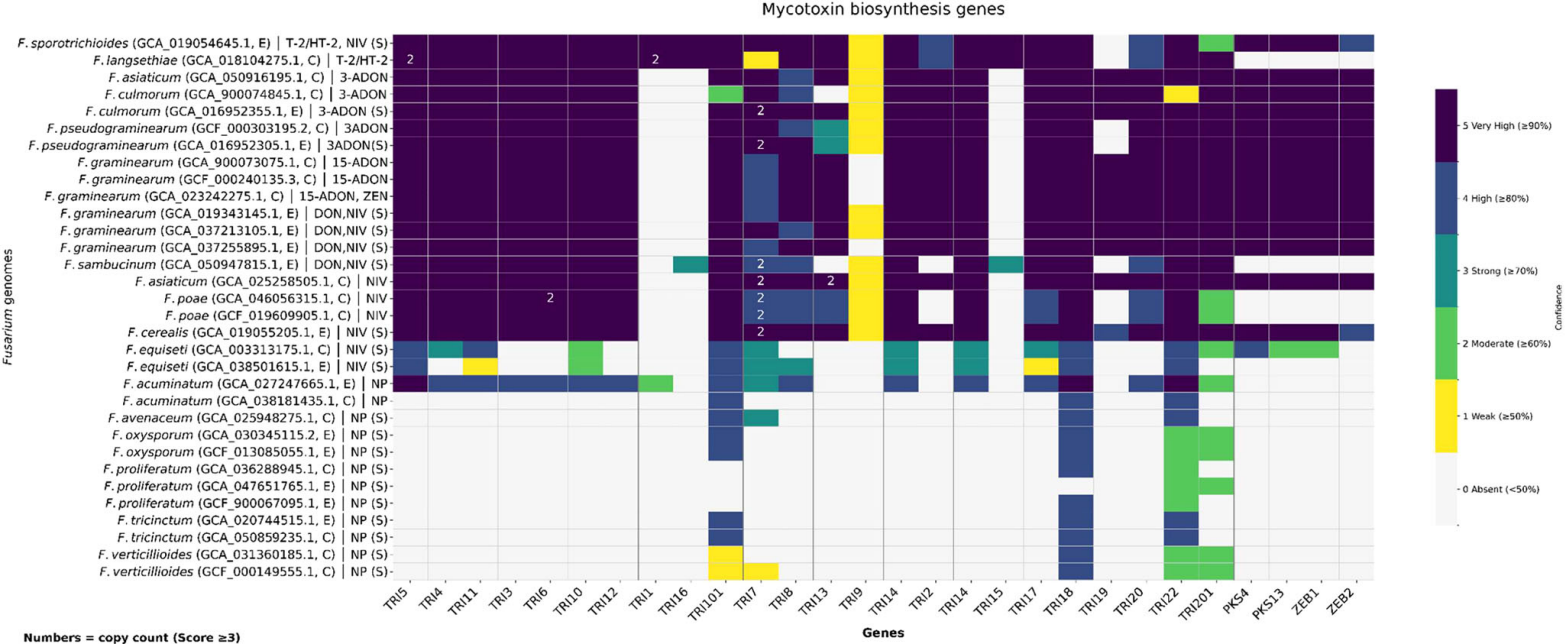
Distribution and confidence of trichothecene (TRI) and zearalenone (ZEN) biosynthesis genes across 31 *Fusarium* genomes. *Fusarium* sp. ordered by chemotype: T‐2/HT‐2, deoxynivalenol (DON), 3‐acetylDON (3‐DON), 15‐acetylDON (15‐ADON), nivalenol (NIV), zearalenone (ZEN), and nonproducers (NP). Genome labels indicate species name, accession number, and genome panel (C = core; E = extended), followed by reported or suspected chemotype (S = inferred based on typical species behavior). Color intensity reflects BLAST‐based confidence scores derived from sequence identity and coverage (see Section 3.3 for details). Numbers within cells indicate gene copy number (if more than a single copy was detected).

Beyond conventional endpoint PCR, the development of quantitative, digital, and multiplex PCR has expanded the sensitivity, quantitative capability, and number of targets that can be assessed in *Fusarium* detection. Quantitative and digital PCR support the estimation or absolute quantification of fungal DNA, while multiplex assays permit the simultaneous detection of multiple *Fusarium* species in wheat (Natarajan et al. 2025; Deng et al. 2024). Table S1 summarizes the principles, advantages, limitations, and applications of legacy and advanced PCR‐based techniques used for Fusarium detection and molecular characterization. A broader methodological comparison of nucleic acid amplification approaches is described by Ndraha et al. (2023) in Table 1 and Figure 1.

**TABLE 1:** Overview of curated *Fusarium* genome panel.

Species name	NCBI accession	Genome assembly level	Reported chemotype	Tier
*F. acuminatum*	GCA_038181435.1	Complete genome	No record	C
	GCA_027247665.1	Contig	No record	E
*F. asiaticum*	GCA_025258505.1	Complete genome	NIV	C
	GCA_050916195.1	Chromosome	3‐ADON	C
*F. avenaceum*	GCA_025948275.1	Complete genome	No record	C
*F. cerealis*	GCA_019055205.1	Contig	No record	E
*F. culmorum*	GCA_900074845.1	Chromosome	3‐ADON	C
	GCA_016952355.1	Complete genome	No record	E
*F. equiseti*	GCA_003313175.1	Contig	TRI and ZEN	C
	GCA_038501615.1	Contig	No record	E
*F. graminearum*	GCA_023242275.1	Chromosome	15‐ADON, ZEN	C
	GCA_900073075.1	Complete genome	15‐ADON	C
	GCF_000240135.3	Chromosome	15‐ADON	C
	GCA_019343145.1	Chromosome	No record	E
	GCA_037213105.1	Complete genome	No record	E
	GCA_037255895.1	Complete genome	No record	E
*F. langsethiae*	GCA_018104275.1	Contig	T‐2/HT‐2	C
*F. oxysporum*	GCA_030345115.2	Complete genome	No record	E
	GCF_013085055.1	Complete genome	No record	E
*F. poae*	GCA_046056315.1	Complete genome	NIV	C
	GCF_019609905.1	Chromosome	NIV	C
*F. proliferatum*	GCA_036288945.1	Complete genome	Enniatin	C
	GCA_047651765.1	Chromosome	No record	E
	GCF_900067095.1	Scaffold	Fusarin and fujikurin	E
*F. pseudograminearum*	GCA_016952305.1	Chromosome	3‐ADON	C
	GCF_000303195.2	Complete genome	No record	E
*F. sambucinum*	GCA_031360185.1	Complete genome	No record	E
*F. sporotrichioides*	GCF_000149555.1	Scaffold	No record	E
*F. tricinctum*	GCA_050947815.1	Contig	No record	E
*F. verticillioides*	GCA_019054645.1	Complete genome	Fumonisin (FB1)	C
	GCA_020744515.1	Chromosome	Fumonisin (FB1)	C

*Note*: Tier indicates the genome selection category used in this study: C (core) represents high‐quality genome assemblies, while E (extended) represents additional assemblies included to broaden species and chemotype coverage.

### 2.3 *Fusarium* Genomic Resources

In parallel to the advancements in PCR‐based molecular detection, the *Fusarium* genomic resources have also improved over the last two decades. The first *F. graminearum* PH‐1 reference genome was one of the first filamentous fungi to have its genome sequenced and assembled using early‐generation short‐read sequencing. The genome was published in 2002 by the Broad Institute's Fungal Genome Initiative (Cuomo et al. 2007). Since then, the availability of *Fusarium* genomes across the genus has expanded at an unprecedented pace, reaching thousands of genomes, and many high‐quality *Fusarium* genomes have been released. Early work was focused on a handful of the most important *Fusarium* species such as *F. oxysporum* f. sp. *lycopersici* (strain 4287) or *F. graminearum*. The past decade has seen the sequencing of multiple isolates per species, revealing extensive variation within species complexes, including subtelomeric diversity, and the presence of lineage‐specific (accessory) chromosomes within complexes such as *F. oxysporum* and *F. solani* (Hoh et al. 2022; Chiara et al. 2015; Y. Zhang et al. 2020). To evaluate the performance of different previously published PCR‐based assays against the novel genomic resources, we performed an in silico check against a set of *Fusarium* genomes, focusing on the species associated with FHB infection.

## 3 Methods

### 3.1 Identifying Published PCR‐Based Assays

A systematic literature review (SLR) search was conducted to identify studies on molecular (PCR‐based) detection of *Fusarium* in wheat, following the PRISMA guidelines (Page et al. 2021). Two major databases, Scopus and Web of Science, were queried using tailored Boolean search strings combining key terms related to *Fusarium*, wheat, PCR primers, and detection methods (e.g., “identification,” “quantification”). The search had no restriction on publication year. In Scopus, the search was done within the title, abstract, and keyword fields. This yielded 117 records on March 21, 2025. The Web of Science search employed a similar strategy using topic, author, and keyword fields, retrieving 164 records. After removing duplicates, 147 unique studies remained for screening.

Titles and abstracts of all retrieved studies were primarily screened by the first author. Articles were included if they investigated *Fusarium* species in wheat using PCR‐based techniques or evaluated primer sets for *Fusarium* detection or quantification. Exclusion criteria included studies that focused on non‐*Fusarium* pathogens, non‐wheat pathogens, or non‐PCR methods. A flowchart summarizing the literature identification, screening, and selection process is provided in Figure S1. Eligible studies underwent full‐text review. Information on primer sequences, target species, amplicon size, PCR conditions, and validation methods was extracted.

### 3.2 Genomes Selection

Upon completion of the identified published PCR‐based assay (Section 3.1), a curated set of 31 *Fusarium* genomes representing 16 species associated with wheat infections was retrieved from the NCBI GenBank database (https://www.ncbi.nlm.nih.gov/genome/) for assessing the previously identified PCR assays. The starting point was the list of species compiled by Alisaac and Mahlein (2023), who reported 16 *Fusarium* and two *Microdochium* species associated with FHB in wheat. From this baseline, five species, *F. semitectum*, *F. sambucinum*, *F. subglutinans*, *Microdochium nivale*, and *Microdochium majus*, could not be included as there were no publicly available genome assemblies for these species (as of September 5, 2025). Conversely, *F. asiaticum* (Sun et al. 2025), *F. langsethiae* (Torp and Nirenberg 2004), and *F. pseudograminearum* (Obanor et al. 2013) were added to the list of genomes as their presence in FHB was reported. Several genome assemblies were available for some of the selected species. As this baseline list included species with differing levels of ecological and epidemiological relevance to FHB of wheat, atypical or opportunistic taxa such as *F. proliferatum*, *F. verticillioides*, and *F. oxysporum* were retained primarily to broaden the taxonomic coverage and assess potential primer cross‐reactivity, rather than to imply equivalent importance as core FHB‐associated pathogens.

Genome completeness was evaluated using BUSCO (Manni et al. 2021) v5.7.1 (Benchmarking Universal Single‐Copy Orthologs) against the sordariomycetes_odb10 lineage dataset (creation date: January 8, 2024; 3817 orthologs). BUSCO was run in the genome mode with default parameters, using miniprot for gene prediction. In addition, the assembly contiguity and sequence quality metrics were assessed using QUAST (Gurevich et al. 2013) v5.2.0. QUAST was run with default parameters on all selected genome assemblies, reporting standard statistics including total assembly size, GC content, contig counts at different length thresholds, N50, L50, auN, and the frequency of ambiguous bases (*N*s per 100 kbp).

For downstream analyses, the genome list was divided into two tiers according to their quality:

(1) **Core panel**: composed of high‐quality assemblies defined by the three criteria: (i) annotated chromosome‐level assemblies, (ii) N50 value more than 3 Mb, and (iii) biological metadata completeness for the sequenced strain, such as documented chemotype (where applicable) and strain origin from a recognized culture collection;
(2) **Extended panel**: composed of assemblies of lower quality, limited annotation, or representing opportunistic/minor pathogens.

While the core panel was defined by explicit criteria, not all species had genomes meeting all three conditions. In these cases, compromises were made to ensure that each major FHB pathogen was represented: (1) where no data were available about the culture collection strains, the best annotated high‐quality assembly was selected; (2) for species with ambiguous or unreported chemotypes, inclusion was justified based on their consistent association with FHB; and (3) where only contig‐ or scaffold‐level assemblies existed for the species of interest, the annotated genome with the least fragmentation was retained.

It should be noted that the higher fragmentation of the genomes from the extended panel may affect the PCR assays’ evaluation (e.g., obscuring detection of off‐target PCR products). However, retaining the extended set of genomes was deemed an appropriate compromise and necessary for inclusion of the broadest possible range of wheat‐associated *Fusarium* species.

Metadata about the selected genomes, including the strain origins, sequencing technologies, chemotype information, associated publications, and genome quality assessment outputs, are provided in Table S2.

### 3.3 BGC Detection Within the Selected Genomes

To assess the distribution and completeness of TRI and ZEN BGCs across the curated *Fusarium* genome panel, a genome‐wide BLASTn analysis was performed using a set of canonical reference sequences as queries. In total, reference seeds representing 28 unique biosynthetic loci were compiled from multiple sources, comprising 24 TRI genes (*Tri1*–*Tri22*, *Tri101*, and *Tri201*) and four ZEN genes (PKS4, PKS13, ZEB1, and ZEB2). Reference sequences were sourced from six *Fusarium* species, combining legacy GenBank accessions and curated, annotated genomes to maximize pathway representation. These included classical trichothecene cluster‐related sequences deposited under the teleomorph name *Gibberella zeae* (e.g., AF336365.2 and AF336366.2), additional TRI gene accessions (e.g., AY217783), and species‐specific sequences extracted from high‐quality reference genomes of *F. graminearum* PH‐1 (ASM24013v3), *F. sporotrichioides* NRRL3299 (GCA_003012315.1), *F. poae* (ASM1960990v1), and *F. langsethiae* (ASM1810427v1).

BLAST searches (BLASTn) were conducted against all genome assemblies using a threshold of ≥50% sequence identity, ≥50% query coverage, and *E*‐value ≤1 × 10^−20^. Nearby and overlapping matches were then processed to reduce redundant hits from fragmented alignments: (1) strand‐aware clustering of high‐scoring pairs within 200 bp was performed to consolidate the fragmented matches, and (2) genomic proximity‐based deduplication was applied to merge overlapping loci on the same contig. Retained loci were assessed using a 5‐level scoring system based on the weighted percentage identity and coverage. The scores were assigned as follows: 5 (≥90% identity and coverage), 4 (80%–90%), 3 (70%–80%), 2 (60%–70%), and 1 (50%–60%). The scoring system was tuned for consistency between the high confidence score and the known chemotypes of the assessed species. Copy number of BGCs was determined by counting nonoverlapping loci per genome, with confident predictions (score ≥3) annotated on heatmaps. Genes were organized by biosynthetic function: core cluster genes, essential external genes, pathway determinants, and chemotype‐specific markers. Genomes were ordered by chemotype classification (Type A, Type B, ZEN, and nonproducers) to reveal biosynthetic pathway relationships. The presence–absence matrices and the copy number data were visualized as heatmaps with colors representing the confidence levels.

### 3.4 In Silico Evaluation of Published Primers for *Fusarium* Detection

#### 3.4.1 Evaluation Against the Curated Set of *Fusarium* Genomes

The primer sequences were extracted from the papers identified during the literature search (see Sections 3.1 and 3.2). The primers’ specificity was evaluated against the previously constructed *Fusarium* genome library (see Section 3.2). Custom Python scripts were developed to perform genome‐wide in silico analysis of primer performance. The source code is publicly available via GitHub (https://github.com/kavithavijeandran/fusarium_amplicon_finder). The script performs three key steps: (1) identification of primer binding sites across all input genomes (FASTA format) with flexible mismatch tolerance (0–5 mismatches per primer), (2) evaluation of all possible primer orientations (including reverse complements), and (3) detection of putative amplicons sizes, retaining only those with ±20% of the lengths reported in source publications. The analysis recorded amplicon metadata, including genomic coordinates, mismatch patterns, and orientation data for primer mismatch and specificity assessment.

Additionally, mismatch bases were recorded according to their position within the primer, with particular attention given to mismatches occurring within the terminal three bases at the 3ʹ end. Primer orientation, including reverse‐complement binding, was accounted for so the 3ʹ end was interpreted relative to the direction of primer extension. Cases where 3ʹ terminal mismatches may affect interpretations are provided in Table S3. From here onward, this analysis will be referred to as in silico analysis.

#### 3.4.2 Primer‐BLAST

To further evaluate potential off‐target amplification beyond the selected set of *Fusarium* genomes, each primer pair was submitted to the NCBI Primer‐BLAST tool (https://www.ncbi.nlm.nih.gov/tools/primer‐blast/), aligning primers against the entire nucleotide (nt) database. The BLAST was configured to include three relevant taxonomic groups—*Fusarium* (taxid:5506), the broader fungal kingdom (taxid:4751), and wheat (*Triticum aestivum*, taxid:4565)—to identify possible false‐positive binding events outside the intended targets. Default BLAST stringency parameters were retained.

#### 3.4.3 Primer Performance Scoring

Primer performance was evaluated using a unified scoring framework that assessed both the in silico check against the curated set of *Fusarium* genomes (*I*‐score) and Primer‐BLAST exclusivity (*B*‐score). Each component was scored on the same 0–2 scale. A score of 2 was assigned when the primer amplified only the intended target with no off‐target amplification. A score of 1 was given when the primer amplified the target but also produced a single off‐target hit, and a score of 0 was assigned for all other cases (i.e., when the primers did not hit the intended target, or the amplicon on target significantly varied from the expected size, or multiple off‐target amplicons were predicted). The final primer score was calculated as the sum of the *I*‐score and *B*‐score and encoded using letters A–E, where A is the highest and E is the lowest score reflecting the overall specificity and suitability for *Fusarium* detection.

### 3.5 Development of the FusaMIQE Framework

Following the new MIQE 2.0 checklist developed in 2025 (Bustin et al. 2025) to promote good practices in quantitative real‐time PCR trials, the FusaMIQE framework was suggested by adapting the requirements to this review context, considering both taxonomy and chemotype characterization.

## 4 Assessment of PCR‐Based Assays for *Fusarium* Molecular Analysis

### 4.1 Curated Panel of Genomes for *Fusarium* Species Associated With Wheat FHB

The curated genome panel composed of 31 assemblies representing 16 *Fusarium* species associated with wheat FHB is summarized in Table 1 (see full version in Table S2). While most of these species are documented FHB pathogens (Alisaac and Mahlein 2023), the sequenced strains originated from diverse hosts including wheat (*n* = 5), maize (*n* = 4), oats (*n* = 4), barley (*n* = 2), and various other plant hosts, reflecting the broad host range of these species and the opportunistic nature of genome sequencing efforts (see Section 3.2). Assemblies were divided into core (*n* = 16) and extended (*n* = 15) panels based on assembly quality, annotation completeness, and strain metadata availability.

BUSCO analysis indicated the high genome completeness across all assemblies, ranging from 98.6% to 99.6% complete single‐copy orthologs (mean: 99.4%; sordariomycetes_odb10 lineage dataset). Assembly contiguity varied: N50 values ranged from 0.49 Mb (*F. cerealis*: GCA_019055205.1) to 9.99 Mb (*F. acuminatum*: GCA_038181435.1). Twenty‐three of the selected genomes were complete at the chromosome level, while eight were contig‐ and scaffold‐level assemblies. Multiple strains were available for nine species. Only 15 genomes were from traceable culture collections. Similarly, only 15 genomes were of known reported chemotypes.

### 4.2 BGC Analysis

The genome‐wide BLASTn analysis identified TRI and ZEN BGC across all selected *Fusarium* genomes. However, the gene composition and confidence level strongly correlated with the previously experimentally determined chemotype classifications (Figure 1).

Type A TRI‐producing species (*F. sporotrichioides* and *F. langsethiae*) showed uniformly high‐confidence detection of early pathway and regulatory TRI genes (*Tri5*, *Tri4*, *Tri6*, *Tri10*, *Tri11*, *Tri12*). However, several genes commonly used as molecular markers in Type B trichothecene diagnostics exhibited reduced confidence or absence. *Tri9* was consistently underdetected in the two former species, while *Tri20* showed reduced confidence relative to the other core genes. In contrast, Type A‐associated genes, including *Tri1*, *Tri16*, *Tri101*, *Tri7*, *Tri18*, and *Tri22*, were detected at high confidence in *F. sporotrichioides* and *F. langsethiae*. Notably, ZEN biosynthesis genes were absent in *F. langsethiae*.

Species known as DON producers (including both 3‐ADON and 15‐ADON chemotypes) displayed highly consistent and near‐complete TRI gene profiles. Thus, *F. asiaticum*, *F. graminearum*, *F. culmorum*, *F. pseudograminearum*, and *F. sambucinum* showed uniformly high‐confidence detection across most core TRI genes. At the same time, *Tri1* and *Tri16* (the Type A‐associated genes) were consistently absent in all these species. Additionally, *Tri19* was absent in both the *F. pseudograminearum* and one *F. sambucinum* assembly, while being retained in other DON producers. ZEN biosynthetic genes were retrieved in all DON‐producing species.

NIV‐producing species exhibited more heterogeneous TRI gene detection patterns. *Fusarium cerealis* and *F. asiaticum* showed high‐confidence detection across the majority of core TRI genes, closely resembling DON‐producing profiles. In contrast, *F. poae* exhibited reduced confidence across several of the core TRI genes, with detection patterns more similar to those observed in Type A trichothecene producers and in *F. equiseti* and *F. acuminatum*. This included partial loss or reduced confidence for genes typically conserved in DON/NIV producers, suggesting a more divergent or rearranged TRI BGC architecture in these species. ZEN biosynthetic genes were inconsistently detected in NIV producers. Genomes classified as nonproducers (NP) lacked high‐confidence detection of most core trichothecene biosynthetic genes. Nevertheless, sporadic detection of sequences similar to individual TRI‐associated genes was observed, most commonly for *Tri101*, *Tri18*, *Tri22*, and *Tri201* as seen in both *F. oxysporum* genomes included in this study.

### 4.3 Primers for Genus‐Level Resolution in *Fusarium* Detection

Two studies reported primer sets intended for *Fusarium* genus‐level screening targeting β‐tubulin and TEF1‐α loci (Table 2). Across these assays, our specificity evaluation indicated limited exclusivity to *Fusarium*, with Primer‐BLAST returning multiple nontarget hits for both primer sets. The βt1/βt2 primers (Pinson‐Gadais et al. 2007) amplified a 296‐bp β‐tubulin fragment but showed low specificity in Primer‐BLAST (98 nontarget taxa), indicating that this region is insufficiently discriminatory for genus‐restricted detection when applied to diverse environmental or food‐associated DNA backgrounds. The nested TEF1‐α assay (Karlsson et al. 2016) demonstrated the best performance among the two, returning only a single nontarget hit (Bionectriaceae sp.) in Primer‐BLAST.

**TABLE 2:** Published primers used for genus‐level resolution for the identification of *Fusarium* species.

Target region	Primer name	Annealing temperature	Expected size (bp)	*Fusarium* specificity	Reference
β‐Tubulin	βt1/βt2	55	296	No (98)	Pinson‐Gadais et al. 2007
TEF1‐α	Fa7/Ra6, Fa/Ra (Nested)	67	550–600	No (1)	Karlsson et al. 2016

The numerical value stated in the *Fusarium* specificity value indicates the number of off‐target results detected from the Primer Blast hits.

### 4.4 Primers for Species‐Level Resolution in *Fusarium* Detection

We identified 19 studies that reported primer sets designed to achieve species‐level resolution for the detection of *Fusarium* species in wheat (Table 3). These assays span over more than two decades and were derived using diverse strategies, including random polymorphic fragment discovery, ribosomal operon targeting, single‐copy housekeeping genes, and other gene regions. Many of these primers were developed under differing taxonomic frameworks and with limited genomic resources available at the time. We evaluated these primer pairs using the unified scoring framework described in Section 3.4.3, and the results are summarized in Table 3. The performance varied widely across primer design strategies, with only a minority of assays demonstrating robust species‐level specificity when tested in silico against the currently available *Fusarium* genomic resources. Of the 53 primer pairs evaluated, six achieved grade A, eight grade B, 14 grade C, 14 grade D, and 11 grade E. The primers’ performance did not show a clear temporal trend with regard to the time of their development. For example, one of the earliest RAPD‐derived assays, Fc01F/Fc01R, for *F. culmorum* (P. Nicholson et al. 1998) still achieved high specificity (grade A), whereas several more recent primers targeting EF1α or tRNA‐Ile loci (Nicolaisen et al. 2009; Deng et al. 2024) showed potential for a substantial cross‐reactivity and had a primer score that ranged from C to E.

**TABLE 3:** Published primers used for species‐level resolution for the identification of *Fusarium* species infecting wheat.

Target species	Primer name	*T*_a_	Expected size (bp)	Primer origin/target region	In silico specificity per primer mismatch*						Primer‐BLAST hits	Primer score	References
					0	1	2	3	4	5			
*F. graminearum*	Fg16F/ Fg16R	62	400–500	RAPD derived	+	+	+	+	+	+	3	B	P. Nicholson et al. 1998
	Fg16NF/Fg16NR	62	280	RAPD derived	1	1	1	1	1	2	3	D	
	Fgr‐F/Fgc‐R	53	500	IGS	4	4	4	6	10	15	6	E	Jurado et al. 2005
	Fu‐4F/Fgram‐R	53	206	tRNA‐Ile	+	2	3	4	4	4	5	D	Deng et al. 2024
	FgramB379fwd/FgramB411rev	60	56	EF1α	1	1	4	4	5	7	8	D	Nicolaisen et al. 2009
	Fgtubf/Fgtubr	59	111	β‐Tubulin	1	4	5	6	7	10	35;2	E	Sanoubar et al. 2015
	UBC85F/UBC85R	61	332	RAPD derived	0	+	2	5	5	12	4	D	Schilling et al. 1996
	SCAR‐Fg1_F/SCAR‐Fg1_R	n.s	315	SRAP‐derived	0	0	0	0	0	+	1	D	X. Zhang et al. 2014
	SCAR‐Fg2_F/SCAR‐Fg2_R	n.s	657	SRAP‐derived	0	0	0	+	+	1	2	D	
	graminearum_MGB‐F/R*	60	n.s.	RAPD derived	1	1	1	1	1	15	3	D	Waalwijk et al., 2004
	COB1/COB2*	60	n.s	COB	0	+	1	2	2	2	3	D	Kulik et al. 2015
*F. culmorum*	Fc01F/Fc01R	62	570	RAPD derived	+	+	+	+	1	14	+	A	P. Nicholson et al. 1998
	175F/430R	58	245	ITS	3	3	3	3	3	5	22;4	E	Mishra et al. 2003
	Fcu‐F/Fgc‐R	54	200	IGS	+	+	+	4	5	9	2	B	Jurado et al. 2005
	FculBF/FculB	58	220	RAPD derived	+	+	+	+	+	10	+	A	Klemsdal and Elen 2006
	FculC561_fwd/FculC614_rev*	60	72	EF1α	1	1	6	8	11	13	7;1	D	Nicolaisen et al. 2009
	OPT18F/OPT18R	55	472	RAPD derived	0	0	+	+	+	5	+	B	Schilling et al. 1996
	culmorum_MGB‐F/R*	60	n.s.	RAPD derived	+	+	+	+	2	15	+	A	Waalwijk et al., 2004
	Fc03/Fc02	62	140	β‐tubulin	+	1	1	1	2	3	2	C	Sanoubar et al. 2015
*F. avenaceum*	FAF1/FAR	58	389	ITS	0	2	2	2	2	2	27;20	D	Mishra et al. 2003
	Fave574_fwd/Fave627_rev*	60	52	EF1α	0	0	1	2	2	2	23;1	D	Nicolaisen et al. 2009
	FA‐ITSF/FA‐ITSR.	59	272	ITS	0	0	0	0	0	20	16	E	Schilling et al. 1996
	avenaceum_MGB‐F/R*	60	n.s.	RAPD derived	+	2	2	2	6	14	1	C	Waalwijk et al., 2004
*F. equiseti*	FEF1/FER1	58	389	ITS	+	+	+	3	3	4	38;21	C	Mishra et al. 2003
	Feq‐F/Feq‐R	66	990	IGS	0	0	0	1	1	15	+	C	Jurado et al. 2005
	FequiB569_fwd/FequiB598*	60	54	EF1α	+	+	+	1	1	5	29	C	Nicolaisen et al. 2009
*F. sporotrichioides*	Fps‐F/Fsp‐R	61	400	IGS	1	1	1	1	2	5	3	D	Jurado et al. 2005
	FspoA18_fwd/FspoA85_rev*	60	94	EF1α	+	+	1	1	1	2	20	D	Nicolaisen et al. 2009
	FspITS2K/P28SL	n.s	288	ITS2 rDNA	+	+	+	+	+	1	2;3	C	Kulik et al. 2004
	Tox5‐1/Tox5‐sporo_R2	56	400	Tri	0	0	+	1	1	6	+	B	L. Niessen and Vogel 2010
*F. poae*	Fps‐F/Fpo‐R	61	400	IGS	0	+	+	+	+	1	+	B	Jurado et al. 2005
	Fp82F /Fp82R	64	220	RAPD derived	+	+	+	+	+	+	—	C	Parry and Nicholson 1996
	FpoaeA51_fwd/FpoaeA98*	60	72	EF1α	+	+	+	+	+	+	1	B	Nicolaisen et al. 2009
	Tox5‐1/Tox5‐poae_R	57	400	Tri	+	+	+	+	+	5	+	A	L. Niessen and Vogel 2010
	poae_1‐F/poae_1‐R	60	n.s.	RAPD derived	0	0	+	+	+	+	—	C	Waalwijk et al., 2004
*F. pseudograminearum*	Fu‐4F/Fpseu‐R	53	482	tRNA‐Ile locus	+	+	+	+	+	7	+	A	Deng et al. 2024
	Fpl‐1 /Fpl‐2	55	523	EF1α	0	0	+	+	1	3	+	B	Aoki and O'Donnell 1999
	FPG_F/FPG_R	65	779	RAPD derived	+	+	4	6	12	15	4	E	Williams et al. 2002
*F. proliferatum*	Fu‐4F/Fprol‐R	53	680	tRNA‐Ile locus	+	+	1	1	2	12	1	C	Deng et al. 2024
	Fpro220_fwd/Fpro270_rev*	60	51	EF1α	+	+	+	+	+	6	11;1	C	Nicolaisen et al. 2009
*F. verticillioides*	Fu‐4F/Fvert‐R	53	963	tRNA‐Ile locus	+	2	2	2	7	15	3	C	Deng et al. 2024
	Fver356_fwd/Fver412_rev*	60	57	EF1α	+	+	+	+	1	13	8	C	Nicolaisen et al. 2009
*F. langsethiae*	F3/R1	65	700	CYP51C	0	1	11	13	15	15	20	E	Fernández‐Ortuño et al. 2010
	FlangA29_fwd/FlangA95_rev*	60	92	EF1α	+	+	1	1	1	1	11	D	Nicolaisen et al. 2009
	Tox5‐1/Tox5‐powd_R	66	400	Tri	+	+	+	+	+	1	1	B	L. Niessen and Vogel 2010
*F. cerealis*	Fcerf1/Fcerr1	65	300	CYP51C	+	+	3	4	4	6	1	C	Fernández‐Ortuño et al. 2010
	FcerPHOF/FcerPHOR*	61	300	Phosphate permease	+	+	+	+	2	2	6	C	Stakheev et al. 2011
*F. tricinctum*	Ftri573_fwd/Ftri630_rev*	60	58	EF1α	0	0	0	0	0	0	16	E	Nicolaisen et al. 2009
	tri1/tri2	65	215	Partial IGS	1	3	3	3	3	6	6	E	Kulik 2008
*F. acuminatum*	FAC‐F/FAC‐R	65	602	RAPD derived	0	0	0	1	15	15	0	E	Williams et al. 2002
*F. asiaticum*	SCAR‐Fa1_F/SCAR‐Fa1_R	n.s	172	SRAP‐derived	0	0	0	0	0	0	0	E	X. Zhang et al. 2014
*F. subglutinans*	Fsub565_fwd/Fsub622A*	60	57	EF1α	0	0	0	0	0	0	37	E	Nicolaisen et al. 2009
*F. avenaceum* / *F. tricinctum*	FTRAVF2/FTRAVR2	65	618	CYP51C	+	+	+	2	2	5	+	A	Fernández‐Ortuño et al. 2010

*Note*: Primer‐BLAST hits indicate the number of *Fusarium* species (first value) and non‐*Fusarium* taxa (second value) identified during Primer‐BLAST evaluation (e.g., 21;2 = 21 *Fusarium* spp. + 2 non‐*Fusarium* taxa). Primer score represents the final cumulative primer score (A–E) integrating both in silico and Primer‐BLAST performance metrics. See details about the scoring in Section 3. Primer names denoted with an asterisk (*) near primer names denote qPCR primers; those without an asterisk are not specified. Under “In silico specificity per primer mismatch*,” symbols indicate outcomes across increasing mismatch thresholds: +, only a species‐specific amplicon of expected size (±20%) for the target species; numeric value, number of nontarget species detected along with the target species.

Abbreviations: n.s., 0, no amplification (no product) observed across the genomes in the panel.

Although mismatch position was not explicitly weighted in the scoring framework, primer template mismatches occurring within the final three bases at the 3ʹ end of either primer were additionally annotated and reported on in Table S3. Primer pairs with stronger performance, particularly those assigned with grades A and B, are summarized in Table 4. However, primer pairs with target–species mismatches occurring within the terminal three bases of the 3ʹ end were excluded from Table 4, as these may have contributed to the overestimation of assay reliability—for example, Tox5‐1/Tox5‐sporo_R2 has been assigned grade B but was not included in Table 4 for this reason.

**TABLE 4:** Summary of higher‐confidence species‐specific *Fusarium* PCR and qPCR primer sets.

Target species	Primer name	Primer score	References
*F. graminearum*	Fg16F/ Fg16R	B	P. Nicholson et al. 1998
*F. culmorum*	Fc01F/Fc01R	A	P. Nicholson et al. 1998
	FculBF/FculB	A	Klemsdal and Elen 2006
	culmorum_MGB‐F/R*	A	Waalwijk et al. 2004
*F. poae*	Tox5‐1/Tox5‐poae_R	A	L. Niessen and Vogel 2010
	FpoaeA51_fwd/FpoaeA98*	B	Nicolaisen et al. 2009
*F. pseudograminearum*	Fu‐4F/Fpseu‐R	A	Deng et al. 2024
*F. langsethiae*	Tox5‐1/Tox5‐powd_R	B	L. Niessen and Vogel 2010

*Note*: Primer scores are based on the combined in silico and Primer‐BLAST evaluation framework used in this review, where A represents the highest confidence category and B indicates good performance with minor limitations. Primer pairs marked with an asterisk (*) indicate qPCR assays. These primer sets should be interpreted as higher‐confidence starting points for assay selection rather than universally validated diagnostic assays.​

#### 4.4.1 RAPD‐Based Assays

RAPD primers were among the earliest developed. Schilling (1996) developed three primer sets, based on RAPD, of which only OPT18F/OPT18R demonstrated reliable species‐level specificity to *F. culmorum* with a primer score of B, while the others showed limited or no discriminatory power due to cross‐reactivity or lack of amplification. The OPTI8F/OPTI8R primer pair was then further optimized by Klemsdal and Elen (2006) in a one‐tube nested PCR assay. Based on our study, the internal primer set (FculBF/FculBR) showed higher species specificity and received a score of A. Parry and Nicholson (1996) also reported an RAPD‐derived primer pair (Fp82F/Fp82R) for *F. poae*, which received our primer score of C, indicating a moderate level of specificity and sensitivity. Similarly, P. Nicholson et al. (1998) identified polymorphic RAPD loci specific to *F. graminearum* and *F. culmorum*, and primer sets Fg16F/Fg16R, Fg16NF/Fg16NR, and Fc01F/Fc01R were designed to target those polymorphic regions. The primer pair Fc01F/Fc01R demonstrated high specificity to *F. culmorum*. Others, like Fg16F/Fg16R, show cross‐reactivity within the *F. graminearum* species complex, particularly with *F. asiaticum* and *F. meridionale*.

Building directly on the loci suggested earlier by P. Nicholson et al. (1998), Waalwijk et al. (2004) redesigned the Fg16F/Fg16R, Fc01F/Fc01R, and Fp82F/Fp82R assays for real‐time qPCR targeting a short internal fragment (<100 bp) derived from the original amplicon. However, despite the reduction in amplicon length and conversion to a qPCR format, the same potential for off‐target detection of *F. asiaticum* and *F. meridionale* for *F. graminearum* was suggested by our in silico assessment, and no amplicon hits were returned for *F. poae*, similar to the Fp82F/R primer pair it was based on. In addition, Waalwijk et al. (2004) reported a *F. avenaceum*‐specific qPCR assay derived from an RAPD fragment (avenaceum_MGB‐F/R). Notably, when evaluated alongside other *F. avenaceum* primers compiled in Table 3, this assay exhibited the highest species specificity, with only a single off‐target hit (*F. acuminatum*).

Other RAPD fragment‐based primer designs FPG_F/FPG_R and FAC‐F/FAC‐R (Williams et al. 2002) lack specificity (PS = E). Another polymorphic fragment‐based primer was designed through Sequence‐Related Amplified Polymorphism (SRAP) analysis. X. Zhang et al. (2014) reported the design of three SRAP‐derived primer pairs for *F. graminearum* and *F. asiaticum*. All three primer pairs demonstrated either high cross‐reactivity or absence of amplification across *Fusarium* genomes, with primer scores ranging from D to E.

#### 4.4.2 Targeting Ribosomal Operon

Mishra et al. (2003) developed a rapid, multiplex‐PCR, gel‐free diagnostic tool using fluorescently labeled PCR primers for the identification of *F. culmorum* (175F/430R), *F. equiseti* (FEF1/FER1), and *F. avenaceum* (FAF1/FAR). The assay targeted sequence variability within the ITS region of nuclear ribosomal DNA (nrDNA), with each primer pair labeled using distinct fluorophores (FAM, Fluorescein, and ROX, respectively). Despite the user‐friendliness and high sensitivity of this assay, in silico evaluation and Primer‐BLAST suggested substantial cross‐reactivity with multiple *Fusarium* species and non‐*Fusarium* taxa (the scoring at a level of ranging between D and E). Kulik et al. (2004) developed a species‐specific PCR assay targeting the ITS2 rDNA region to detect *F. sporotrichioides*, which also demonstrated potential issues with specificity, showing cross‐reactivity (from 2 mismatches in primers onward) with multiple *Fusarium* species and nontarget fungi, including *Alternaria alternata*.

The highly variable intergenic spacer (IGS) region, which is located between the 28S and 18S rRNA genes, was also targeted for the development of *Fusarium* species‐specific primers. Jurado et al. (2005) reported five primer pairs to detect specific *Fusarium* spp. The primer pairs Feq‐F/Feq‐R (*F. equiseti*) and Fps‐F/Fpo‐R (*F. poae*) demonstrated strong specificity in our in silico checks. In contrast, IGS primers designed for *F. graminearum* and *F. culmorum* exhibited cross‐reactivity primarily within the *Fusarium graminearum* species complex (FGSC) members. The remaining three assays for *F. graminearum*, *F. culmorum*, and *F. sporotrichioides* showed cross‐reactivity with other species within the genus, such as *F. asiaticum*, *F. langsethiae*, and *F. meridionale*. Kulik (2008) reported a primer pair (tri1/tri2) designed from the IGS region for the detection of *F. tricinctum*. In our analysis, we demonstrated cross‐reactivity with several other *Fusarium* spp. including *F. acuminatum*, *F. avenaceum*, and *F. heterosporum*.

#### 4.4.3 Single‐Copy Housekeeping Genes

Aoki and O'Donnell (1999) reported a primer pair for the TEF‐1α region to detect *F. pseudograminearum* (formerly known as group 1 *F. graminearum*), the specificity of which was confirmed by our analysis. Nicolaisen et al. (2009) designed 11 species‐specific primer pairs targeting the EF1α gene for the detection of common *Fusarium* species in cereals. Primer‐BLAST and in silico analysis showed that only the *F. poae*‐specific primer (FpoaeA51/FpoaeA98) showed consistent species specificity across both analyses. The remaining primers either did not amplify or exhibited cross‐reactivity with multiple *Fusarium* species such as *F. asiaticum*, *F. meridionale*, and *F. acuminatum*. Sanoubar et al. (2015) reported *F. graminearum*‐specific primers Fgtubf/Fgtubr targeting the β‐tubulin gene, aiming to provide accurate species specifically in wheat‐derived samples. Our analysis suggested a broad cross‐reactivity of this assay with 35 *Fusarium* species, including *F. asiaticum*, *F. cerealis*, and *F. culmorum*, as well as a non‐*Fusarium* pathogen (*Sclerotinia sclerotium*).

#### 4.4.4 Other Genes Applied for *Fusarium* Species Detection

L. Niessen et al. (2004) developed a series of species‐specific primer sets targeting the *Tri5* gene, which encodes the trichodiene synthase. Using sequence alignments of *Tri5* from various type A trichothecene‐producing *Fusarium* species, they designed a universal forward primer (Tox5‐1) paired with species‐specific reverse primers for *F. poae*, *F. sporotrichioides*, and *F. langsethiae*. Two of the three reported primer pairs, *F. poae* (Tox5‐1/Tox5‐poae_R) and *F. sporotrichioides* (Tox5‐1/Tox5‐sporo_R2), demonstrated species‐specific amplification in our in silico checks (up to 4 mismatches in primers) and returned no significant off‐targets in Primer‐BLAST, indicating high specificity. In contrast, the primer for *F. langsethiae* (Tox5‐1/Tox5‐powd_R) did not amplify in silico and showed cross‐reactivity with *F. sporotrichioides*.

The phosphate permease gene is a single‐copy protein‐coding gene with sufficient interspecies variability. It was used by Stakheev et al. (2013) to develop a primer pair for detecting *F. cerealis*. While our in silico analysis indicated high specificity within wheat‐associated *Fusarium* spp., the broader Primer‐BLAST searches revealed potential cross‐reactivity with six other *Fusarium* species, including *F. graminearum*, *F. boothii*, and *F. meridionale*.

The CYP51C gene has also been reported as a taxonomically informative one to resolve among *Fusarium* species. Fernández‐Ortuño et al. (2010) designed two primer pairs targeting this gene: F3/R1 for the detection of *F. langsethiae*, and Fcerf1/Fcerr1 for *F. cerealis*. However, our in silico analysis showed that F3/R1 failed to amplify the target (even when allowing for several mismatches in primers) and exhibited potential cross‐reactivity with up to 20 *Fusarium* species in Primer‐BLAST. The Fcerf1/Fcerr1 primer pair produced the expected amplicon but also showed off‐target product with *F. culmorum*, indicating limited specificity under relaxed binding conditions (with 2 mismatches in primers).

#### 4.4.5 Multiplex Assay Targeting tRNA‐Ile Gene

More recently, Deng et al. (2024) developed a multiplex PCR assay for detecting *F. graminearum*, *F. pseudograminearum*, *F. proliferatum*, and *F. verticillioides*, using a conserved region within a tRNA‐Ile gene for a universal forward primer and species‐specific reverse primers. The assay reported a detection limit of 100 pg/µL of gDNA. Our in silico analysis showed high specificity for *F. pseudograminearum*, while primers for *F. graminearum*, *F. proliferatum*, and *F. verticillioides* displayed cross‐reactivity with other *Fusarium* species, especially within the *F. fujikuroi* species complex.

The main BGCs that code for genes required for the synthesis of trichothecenes (*TRI* cluster) and ZEN (*ZEB* and *PKS*) have been the key targets for the development of PCR‐based assays for chemotype prediction within the *Fusarium* spp. Table 5 lists primer sets designed to target genes within both of these BGCs and compares the chemotype predictions based on in silico PCR results with the previously reported chemotypes in our curated *Fusarium* genome panel.

**TABLE 5:** Published primers used for chemotype‐level resolution of *Fusarium* isolates infecting wheat.

Target gene	Primer name	*T*_a_	Expected size (bp)	Corresponding chemotype	In silico specificity per primer mismatch*						Primer‐BLAST hits	References
					0	1	2	3	4	5		
*Tri5*	Tox5‐1/Tox5‐2	60	650	TRI (All)	+	+	1	1	1	1	1	M. L. Niessen and Vogel 1998
	Tri‐5F/Tri‐5R	62	544	TRI (All)	+	+	1	1	1	1	3	Sanoubar et al. 2015
*Tri4*	T4F1506/T4EndR2	54	550	TRI Type A	+	+	+	1	2	2	+	P. C. Nicholson et al. 2004
	Tri4BF/Tri4BR	62	450	TRI Type B	+	+	+	1	2	2	+	
	Tri1434/Tri1433	60	600	TRI	+	+	+	+	1	1	+	Lacmanová et al. 2009
*Tri3*	Tri3F971/ Tri3R1679	53	354	Tri3 15‐ADON	0	0	+	2	2	2	2	Quarta et al. 2006
	Tri3F1325/ Tri3R1679	53	708	Tri3 3‐ADON	+	+	+	+	+	4	1	
	3CON/3NA	52	840	Tri3 NIV	+	+	2	3	3	4	1	Starkey et al. 2007
	3CON/3D15A	52	610	Tri3 15‐ADON	+	+	1	1	3	4	+	
	3CON/3D3A	52	243	Tri3 3‐ADON	+	+	+	+	1	4	+	
*Tri6*	Tri6‐F/Tri6‐R	60	541	TRI (All)	+	+	+	+	1	1	1	Neera and Murali 2021
*Tri12*	12CON/12NF	52	840	NIV	+	+	1	2	4	4	+	Starkey et al. 2007
	12CON/15‐15F	52	670	15‐ADON	+	+	+	1	4	4	+	
	12CON/12‐3F	52	410	3‐ADON	+	+	1	1	2	4	1	
*Tri7*	Tri7F/Tri7NIV	60	436	NIV	+	+	+	+	1	2	+	Chandler et al. 2003
	Tri7F/Tri7DON	60	458–535	DON	+	+	+	+	+	1		
*Tri8*	tri8‐F/tri8‐R	60	144	Type A (T‐2)	0	+	+	1	4	4	+	Neera and Murali 2021
*Tri13*	Tri13NIVF/Tri13R	58	312	NIV	+	+	+	+	1	4	+	Chandler et al. 2003
	Tri13F/Tri13DONR	59	282	DON	0	+	1	2	2	2	+	
	Tri13P1/Tri13P2	58	859	NIV	+	1	1	1	2	4	+	Amarasinghe et al. 2011
			644	3‐ADON	0	0	1	1	1	1		
			583	15‐ADON	0	0	1	1	1	1		
*PKS4*	Pks4‐F/pks4‐R	60	743	ZEN	+	+	+	+	1	1	+	Neera and Murali 2021

*Note* *: T*_a_ = annealing temperature. A numeric value indicates the number of nontarget chemotypes amplified in addition to the target chemotype, and “0” indicates no amplification across the genomes included in the panel. For Primer‐BLAST evaluation, “+” indicates specificity to the genus *Fusarium*, whereas numeric values indicate the number of non‐*Fusarium* taxa with predicted amplicons. Type A and Type B refer to the major structural classes of trichothecenes. Under in silico specificity per primer mismatch thresholds, “+” indicates amplification of only the expected target chemotype within ±20% of the expected amplicon size.

Abbreviations: 15‐ADON, 15‐acetyldeoxynivalenol; 3‐ADON, 3‐acetyldeoxynivalenol; DON, deoxynivalenol; NIV, nivalenol; T‐2, T‐2 toxin; TRI, trichothecene; ZEN, zearalenone.

#### 4.4.6 Trichothecene (TRI) Cluster Assays

The TRI genes *Tri5*, *Tri4*, *Tri6*, and *Tri10‐12* are core components of the TRI BGCs and are frequently used as molecular markers for TRI‐producing *Fusarium* species. Thus, several PCR assays had been developed for the detection of TRI‐producing *Fusarium* species to serve as a proxy for TRI biosynthesis capacity. First, M. L. Niessen and Vogel (1998) targeted a ∼650‐bp fragment of the *Tri5* gene (trichodiene synthase), then Sanoubar et al. (2015) applied a related *Tri5*‐based PCR assay, generating a 544‐bp amplicon to screen for TRI biosynthetic potential. For both assays, in silico analysis showed comparable good performance: at <2 mismatches in primers, the expected amplicon was consistently detected in species associated with 3‐ADON, 15‐ADON, and T‐2/HT‐2 chemotypes, indicating broad coverage of TRI‐producing lineages. The Primer‐BLAST analysis identified a limited number of non‐TRI‐producer (and non‐*Fusarium*) off‐targets that may confound the *Fusarium* chemotype detection. The off‐targets included *Tricholoma matsutake* for the Tox5‐1/Tox5‐2 primer pair and *Microdochium bolleyi*, *Stagonosporopsis rhizophila*, and *T. matsutake* for the Tri‐5F/Tri‐5R assay, of which only *M. bolleyi* is ecologically associated with wheat but is not a TRI producer. Exploring whether these hits for the core TRI gene amplicons are artifacts of spurious genomic sequence similarities or have relevance to the biochemistry of these species is beyond the scope of this review. However, such off‐target amplicons may interfere with the prediction of the *Fusarium* species chemotypes.

Designing a PCR assay for chemotype prediction, P. C. Nicholson et al. (2004) leveraged the sequence divergence between Type A and Type B trichothecene producers at the *Tri4* locus. This resulted in two pathway‐stratified PCR assays, including T4F1506/T4EndR2 for Type A and Tri4BF/Tri4BR for Type B producers. While both assays were specific to the *Fusarium* genus (Primer‐BLAST) in our in silico check, both primer sets demonstrated robust chemotype discrimination in the evaluated genome panel. Lacmanová et al. (2009) have adapted the primers designed by McCormick et al. (2006) based on the *Tri4* gene to detect TRI‐biosynthesis potential. The primer pair Tri1434/Tri1433 (which amplifies an internal ∼588–590 bp fragment of *Tri4*) showed expected in silico amplicons with perfect primer matches (0–1 mismatch per primer set) across all Type‐B trichothecene producers within our *Fusarium* genomes panel. Conversely, Type A producers only exhibited expected amplicons if 2 or 3 primer mismatches were allowed, no valid binding sites were detected in nonproducing *Fusarium* species, and Primer‐BLAST returned no non‐*Fusarium* hits for the Tri1434/Tri1433 assay.

Neera and Murali (2021) developed a multiplex PCR assay targeting TRI‐ (541 bp), T‐2‐ (144 bp), and ZEN‐producing (743 bp) *Fusarium* species. The primer pair tri6‐F/tri6‐R (Ramana et al. 2011; adopted by Neera and Murali 2021) that amplifies a fragment of *Tri6* and the primer pair tri‐8‐F/tri‐8‐R that targets *Tri8* both showed strong performance in our in silico validation, producing expected amplicons with perfect primer matches across the intended targets. Similarly, both assays demonstrated genus‐level specificity in the Primer‐BLAST analysis.

While the presence of core TRI genes is the key for the detection of trichothecene biosynthetic potential in general, more detailed chemotype differentiation within Type B trichothecene producers relies on allelic polymorphisms and gene disruptions within specific TRI loci rather than the simple presence of the relevant gene. Several PCR‐based assays have targeted polymorphisms in chemotype‐associated TRI genes, including *Tri3*, *Tri12*, *Tri7*, and *Tri13*, to discriminate between 3‐ADON, 15‐ADON, and NIV chemotypes. Quarta et al. (2006) developed a multiplex assay exploiting sequence polymorphisms in *Tri3* to distinguish 3‐ADON and 15‐ADON chemotypes using a shared reverse primer (Tri3R1679) and chemotype‐specific forward primers (Tri3F1325 for 3‐ADON; Tri3F971 for 15‐ADON), generating amplicons of 708 and 354 bp, respectively. Both assays showed effective chemotype discrimination in our in silico check against the curated gene panel. However, the Primer‐BLAST analysis identified a limited number of non‐*Fusarium* matches, including *Rhizophagus irregularis*, *Tricholoma hemisulphureum*, *Saccharomyces bayanus*, and *S. uvarum*. To our knowledge, these species have not been reported as occurring on wheat spikes.

Another PCR‐based chemotyping test system for members of FGSC was developed by Ward et al. (2002) and later validated by Starkey et al. (2007). The approach employed two multiplex PCR assays targeting polymorphic regions of the *Tri3* and *Tri12* genes. The primers targeting the *Tri3* gene served as the primary diagnostic, while *Tri12* targeting was used as a confirmatory step. These assays leveraged the sequence differences among 3‐ADON, 15‐ADON, and NIV chemotypes to produce distinct amplicon sizes for each group, enabling simultaneous detection and differentiation of chemotypes. The in silico analysis against the genome panel confirmed a high specificity of this test system for Type B TRI producers, confirming the validity of Starkey et al.’s (2007) approach to use allelic polymorphisms in *TRI3* for chemotype discrimination against the current genomic resources within the FGSC.

Chandler et al. (2003) developed a PCR‐based test system targeting the *Tri13* and *Tri7* genes to distinguish between NIV and DON chemotypes in *F. graminearum*, *F. culmorum*, and *F. cerealis* strains. The test system included two primer sets targeting *Tri13*, which were designed for detecting a deletion within the *Tri13* gene that differs between DON‐ and NIV‐producing *Fusarium* strains. Indeed, NIV producers have a functional *Tri13* gene, while DON producers have deletions at positions 178, 61, and 37, leading to disruption of Tri13 activity. These deletions were reported to be highly conserved across DON chemotypes. The Tri13NIV‐specific primers of Chandler et al. (2003) test system produced a 312‐bp amplicon exclusively in NIV producers, while Tri13DON‐specific primers generated a 282‐bp amplicon in DON producers. This was complemented by the *Tri7*‐targeting assays that exploited insertions or deletions within the *Tri7* locus, as confirmatory markers. When assessed in silico against the curated set of *Fusarium* genomes, the *Tri13*‐based assays were robust for the intended purposes. However, the *Tri7‐*based confirmatory step was only specific for DON, but not for NIV. All the assays were specific to the *Fusarium* genus, without producing any additional amplicons in the Primer‐BLAST check.

#### 4.4.7 ZEN BGC Assays

In contrast to the TRI chemotyping, molecular detection of ZEN production has largely focused on presence/absence screening of the ZEN BGC, rather than fine‐scale polymorphism analysis. The PKS4 gene, encoding the polyketide synthase essential for ZEN biosynthesis, has been the most widely adopted marker for ZEN‐producing *Fusarium* species. The Pks4‐F/pks4‐R primer set reported by Neera and Murali (2021) showed a very good performance in our tests: it consistently generated the expected 743‐bp amplicon across ZEN‐producing genomes in our curated *Fusarium* genomes. Primer‐BLAST analysis also indicated high genus‐level specificity, with no wheat‐associated non‐*Fusarium* off‐targets detected.

### 4.5 qPCR for *Fusarium* Detection and Quantification

qPCR enables the sensitive detection and quantification of *Fusarium* DNA in wheat, providing a means to estimate fungal biomass complementary to the plate‐counting diagnostics. However, reported assay sensitivity, specificity, and diagnostic scope vary substantially across studies, reflecting differences in primer design, target selection, calibration strategies, and reporting practices. The introduction of Minimum Information for Publication of Quantitative Real‐Time PCR Experiments (MIQE) guidelines in 2009 (Bustin et al. 2009) marked a critical step toward establishing a standard for qPCR assay's validation and reporting. While the MIQE guidelines provide a comprehensive framework for the reporting of qPCR experiments across diverse biological applications, not all categories are equally informative or consistently applicable to pathogen diagnostics in complex plant matrices.

To enable a focused and biologically meaningful assessment of *Fusarium* qPCR assays in wheat, a *Fusarium*‐tailored MIQE and MIQE 2.0 (Bustin et al. 2025) checklist was established, hereafter referred to as FusaMIQE (Table 6), that retains parameters with the greatest impact on assay interpretability, reproducibility, and interstudy comparability. Specifically, the original nine MIQE categories were consolidated into six core domains—study design; sample and nucleic acid quality; primer and target information; qPCR protocol; data analysis; and reporting and reproducibility—based on relevance to wheat‐associated *Fusarium* diagnostics. Categories relating primarily to instrument calibration, experimental workflow logistics, or postanalytical normalization strategies were included within broader categories or excluded where they did not materially influence the biological interpretation of *Fusarium* DNA quantification. Parameters designated as Essential were considered critical for assessing assay validity and biological meaning, whereas Desirable parameters were those that improve transparency, robustness, and cross‐study comparability but are context dependent or inconsistently reported in the current literature.

**TABLE 6:** QPCR FusaMIQE checklist for authors, reviewers, and editors.

Category	Parameters to report	Description	Rank
Study design	Experimental and control groups	Definition of sample groups analyzed (e.g., FHB‐positive vs. FHB‐negative wheat samples; mock DNA communities, reference strains)	E
	Number of biological replicates	Number of independent biological samples analyzed (e.g., field isolates, grain samples, or DNA extractions)	E
	Technical replicates	Minimum of two qPCR replicates per sample recommended; report mean and variability.	E
Sample and nucleic acid quality	Sample source and type	Description of sample materials analyzed (e.g., grains, wheat heads, pure culture, or soil)	E
	Storage conditions	Storage duration, temperature, or usage of preservatives	E
	Extraction method	Protocol name, kit manufacturer and catalog number, amount of material processed, and any modifications. Different methods selectively enrich or deplete fragment sizes.	E
	DNA quality metrics	Concentration (ng/µL) and at least one purity indicator (A260/280; A260/230 for grain extracts prone to polyphenol contamination). Gel electrophoresis or fluorometry to assess integrity.	E
	PCR inhibition check	Dilution series testing (minimum undiluted, 1:5, and 1:10) to detect efficiency shifts indicative of inhibition. An internal amplification control (IAC) is strongly recommended in grain and flour matrices where phenolic compounds, fatty acids, and starch are documented inhibitors.	D
	Exogenous spike‐in	Addition of a heterologous nucleic acid (e.g., a synthetic or nontarget organism DNA) of known quantity prior to extraction. Monitors extraction efficiency, sample‐to‐sample variability, and independent confirmation of inhibition beyond the dilution test.	D
Primer and target information	Target gene/cluster	Genomic locus targeted (e.g., *Tri5*, *Tri4*, *ZEB1*, *PKS4*); provide NCBI accession number for reference sequence used in primer design or the origin papers reporting it	E
	Amplicon length and position	Amplicon size in base pairs and genomic location, including exon–intron boundaries where relevant	E
	Primer sequences and source	Primer (and probe, if applicable) sequence or publication	E
	Empirical validation	Experimental confirmation of using agarose gel electrophoresis, melt curve analysis (for SYBR Green assays), or probe‐based specificity confirmation (for TaqMan assays)	E
	Bioinformatics design tools	Software and version (or database query date) used for primer design and in silico specificity screening (e.g., Primer‐BLAST, Primer3Plus)	D
	Chemotype coverage*	Expected coverage of toxin chemotypes (e.g., DON, NIV, T‐2/HT‐2, or ZEN)	D
	In silico validation	BLAST against fungi, wheat, and wheat ecosystem organisms	E
qPCR protocol	Reaction conditions	Complete reaction composition, including polymerase, Mg^2+^ concentrations, dNTPs, primer and probe concentration, and reaction volume	E
	Thermocycler model and cycling profile	Instrument model and full thermal cycling program	E
	Standard material identity and quantification	Identity of calibration standard: genomic DNA from a verified strain, synthetic construct, or plasmid; the method used to quantify the standard must be stated	E
	PCR efficiency, slope, R^2^,	Amplification efficiency (90%–110% acceptable range), slope, *R* ^2^ (≥0.98); report confidence interval of efficiency estimate. The standard curve should span four or more orders of magnitude with ≥3 replicates per dilution point.	E
	Detection and quantification limits (LOD and LLOQ)	Distinguish the limit of detection (LOD: smallest amount reliably detected, typically ≥3 copies at 95% probability) from the lower limit of quantification (LLOQ: smallest amount that can be accurately quantified, typically ∼10 copies per reaction). Also, report the upper limit of quantification (ULOQ) to define the working range. Reporting only LOD is insufficient for quantitative claims.	E
	No‐template controls (NTCs)	NTCs must be included in every run. Report whether amplification occurred and, if so, the Cq value. A sample Cq must precede the NTC Cq by ≥5 cycles to be considered positive. Results from a contaminated run should not be reported.	E
	Positive control	Verified *Fusarium* genomic DNA from a strain of known species identity and chemotype (where relevant), run alongside experimental samples in every run	E
	Extraction/field blank	An extraction blank (reagents processed through the full extraction without biological material) must be included to detect reagent or laboratory contamination.	E
Data analysis	Quantification cycle (Cq/Ct) determination method	Methods used to determine the quantification cycle (Cq/Ct), including software and threshold‐setting approach.	E
	Quantification model	Absolute quantification via a standard curve is recommended. Cq values must be converted to efficiency‐corrected target quantities before reporting quantitative results. The ΔCq method is acceptable for relative comparisons provided PCR efficiencies are shown to be equivalent. The ΔΔCq method is not recommended.	E
	Biological versus technical variation	Reporting of variability between biological and technical replicates (e.g., standard deviation, or coefficient of variation)	D
	Raw data availability	Availability of raw qPCR output data (e.g., Cq tables or RDML files)	D
Reporting and reproducibility	Interpretation context	Clear distinction between presence/absence detection and quantification interpretation	E
	Chemotype inference limits*	Discussion of possible limitations in chemotype prediction	E
	Primer metadata availability	Deposition or accessibility of primer and probe metadata	D

*Note*: This checklist is adapted from the MIQE guidelines (Bustin et al. 2009, 2025) and tailored to *Fusarium* diagnostics in wheat‐based matrices. Importance rank reflects the relative impact of each parameter on assay interpretability and reproducibility: E (Essential) indicates parameters considered critical for the validity and comparability of qPCR results, while D (Desirable) indicates parameters that strengthen assay robustness and transparency but are less frequently reported or context dependent. Parameters marked with an asterisk (*) are applicable only when context dependent.

Abbreviation: FHB, fusarium head blight.

Using the *Fusarium*‐tailored *Fusa*MIQE checklist (Table 6), reporting completeness was evaluated for 16 wheat‐related *Fusarium* qPCR studies spanning 2001–2023 (Table S4). Nearly all studies clearly reported the core qPCR conditions such as target gene/cluster, primer sequences, empirical validation, reaction conditions, and cycling profiles. Study design reporting was also generally complete, with experimental/control structure along with biological replicates reported in most studies. In contrast, core‐analytical metadata were underreported. Storage conditions, for example, were only clearly reported in seven out of 16 studies, while DNA quality metrics were fully reported in only six out of 16 studies. Indeed, many of the studies reported quantification of DNA and indicated assessing quality without providing quality data. Inhibition assessment was also reported more often but remained inconsistent. Raw data availability was almost absent (2/16 reported), limiting reproducibility and cross‐study benchmarking.

## 5 Discussion

### 5.1 Genome Selection and Dataset Limitations

The reliability of in silico primer evaluation in this study was strongly influenced by genome selection, assembly quality, and metadata completeness. The high genome completeness observed across all assemblies (98.6%–99.6% BUSCO) is the first key point supporting the suitability of the curated dataset used for primer specificity evaluation in our study. However, substantial variation in assembly contiguity and metadata completeness (source of strain and chemotype records) necessitated a two‐tier approach to genome curation. Assembly contiguity broadly reflected sequencing strategy, with chromosome‐level assemblies predominantly generated using long‐read sequencing technologies (Pacific Biosciences or Oxford Nanopore Technologies), whereas contig‐ and scaffold‐level assemblies were largely derived from short‐read Illumina data. Importantly, this limitation, genome fragmentation, may impose downstream implications for in silico primer evaluations, as fragmented assemblies and incomplete genomic representations may obscure potential off‐target binding sites, leading to an overestimation of primer specificity. As a result, in silico predictions should be interpreted cautiously in highly fragmented genome assemblies, as they may not fully reflect real‐life PCR behavior.

Metadata completeness varied substantially across the genomes. Only 10 of the 16 core‐panel assemblies were derived from strains deposited in recognized culture collections, and chemotype information was available for only 15 genomes. Applying all three “core” panel criteria stringently (annotated assembly, N50 >3 Mb, authenticated strain with documented chemotype) would have reduced the dataset to fewer than 10 genomes, disproportionately excluding several major FHB‐associated species. To balance quality with taxonomic breadth, the dataset was divided into “core” (*n* = 16) and “extended” (*n* = 15) panels. The core panel prioritized assemblies meeting at least two of the three quality criteria and representing major FHB pathogens, while the extended panel included lower‐quality assemblies or opportunistic species to assess primer cross‐reactivity and intraspecific variation. Critically, primer performance scoring (Section 3.4.3) relied exclusively on the “core” panel results, with “extended” panel data serving to flag potential off‐target amplification rather than contributing to primary specificity assessments. Species representation within the dataset was uneven, reflecting public genome availability rather than ecological prevalence. In particular, *F. graminearum* (*n* = 6) was overrepresented due to its central role in FHB epidemiology and the numerous available high‐quality genomes.

### 5.2 BGC Completeness Analysis

The genome‐wide analysis of TRI and ZEN BGCs completeness across *Fusarium* species demonstrated a strong BGC architecture and experimentally defined chemotype classification across the curated *Fusarium* genome panel. Type B TRI producers displayed largely complete and coherent TRI gene profiles, whereas a higher variability was observed among other chemotypes. For several TRI genes, particularly those exhibiting strong allelic divergence between Type‐A and Type‐B TRI producers, limited reference diversity may reduce detection sensitivity for divergent lineages. This apparent variability is consistent with the modular evolution of TRI BGC, in which accessory loci are more prone to lineage‐specific degeneration, truncation, or functional repurposing, while core genes remain conserved (Proctor et al. 2018).

The *Tri9* locus clearly illustrates these limitations. Publicly available *Tri9* reference sequences are sparse and frequently partial, and the gene itself is a very short, poorly characterized open reading frame (ORF) (≈129 bp / 43 aa) with poorly characterized function (Ward et al. 2002). In the present analysis, *Tri9* produced low‐confidence matches across genomes, likely reflecting annotation inconsistencies and assembly limitations rather than the true absence of the locus. The presence of pseudogenes within the TRI cluster further complicates the interpretation of PCR‐based diagnostics. Several loci, including *Tri7* and *Tri13*, exhibit lineage‐specific functionality, where functional alleles are associated with specific chemotypes, while nonfunctional or truncated variants are present in DON producers. Similarly, *Tri16* has been implicated in T‐2 toxin producers but may be absent or nonfunctional in other lineages.

These observations also highlight that not all genes within the BGC are suited as diagnostic targets. In particular, the distinction between causal genes directly involved in toxin biosynthesis and accessory or proxy loci becomes critical when developing PCR‐based chemotype assays, as reliance on noncausal markers may lead to misleading interpretations of biosynthetic potential. Overall, this BGC completeness analysis provides an important genomic context for interpreting PCR‐based chemotype diagnostics. It demonstrates that gene presence–absence patterns must be interpreted cautiously, particularly for short or poorly characterized loci.

### 5.3 Genus‐Level Detection and Universal Primers

Genus‐level detection of *Fusarium* remains an important component of PCR‐based molecular surveillance strategies, particularly in contexts where rapid screening is prioritized over precise species resolution. In many practical applications, including routine grain testing, large‐scale surveillance surveys, and the analysis of processed food commodities, DNA quality may be compromised. Mechanical processing and thermal treatments can fragment template DNA and reduce the quantity available for amplification, while co‐extracted matrix compounds such as phenolics, fatty acids, and starch could further constrain extraction efficiency and may inhibit downstream PCR. In these settings, genus‐level assays provide a cost‐effective and high‐throughput means of estimating overall *Fusarium* pressure and allowing samples of concern to be flagged for downstream analysis. However, broad‐range primers may sacrifice taxonomic resolution. From the findings of this paper, the two wheat‐relevant *Fusarium* genus‐specific primer sets βt1/βt2 (Pinson‐Gadais et al. 2007) and Fa/Ra (Karlsson et al. 2016) both exhibited cross‐reactivity with non‐*Fusarium* taxa, though at markedly different degrees (98 and two off‐target Primer‐BLAST hits, respectively).

### 5.4 Primers for Species‐Level Resolution in *Fusarium* Detection

Reliable species‐level resolution using single‐locus PCR assays remains inherently challenging in *Fusarium*, particularly among recently diverged species complexes (e.g., FGSC, *F. incarnatum–equiseti* species complex [FIESC], and *F. oxysporum* species complex [FOSC]). In these groups, species boundaries are difficult to resolve using a single genetic marker due to high sequence similarity and ongoing evolutionary processes, including incomplete lineage sorting and gene flow (Bugingo et al. 2025). Our systematic evaluation confirms these fundamental constraints, where robust species‐specific resolution was achieved for only a limited subset of the assays assessed.

The variable performance of early RAPD‐derived markers reflects their design limitation and the major taxonomic reclassification of the genus that happened after the development of these markers (Summerell 2019). Despite these limitations, several RAPD markers such as Fg16F/Fg16R have remained in diagnostic use due to their empirical effectiveness. The OPTI8F/OPTI8R assay for *F. culmorum* (Schilling 1996) exemplifies the distinction between locus specificity and primer optimization. Although the primer set requires relaxed mismatch tolerance to amplify, the region amplified by these primers is a true species‐specific locus supported by BLAST (Sequence ID: AY880844.1), indicating that reduced assay performance stems from primer placement rather than target choice.

Primers targeting the ribosomal operon region (ITS, ITS2, and IGS) consistently underperformed for species‐level resolution in *Fusarium*. This observation aligns with the established consensus that ribosomal markers often fail to distinguish between phylogenetically distinct species as they share identical or near‐identical ITS rDNA alleles (O'Donnell et al. 2015). While these multicopy loci provide high sensitivity, they lacked sufficient discriminatory power within *Fusarium*, particularly among members of FGSC and FIESC. Even primers designed within highly variable IGS regions frequently cross‐amplified phylogenetically adjacent species. This observation aligns with previous assessments showing ribosomal markers are often insufficient for resolving closely related *Fusarium* species due to high interspecific sequence conservation (Geiser et al. 2004).

Single‐copy housekeeping genes such as EF1‐α and β‐tubulin showed mixed performances. Although these loci are widely used in multilocus phylogenetics, short diagnostic amplicons extracted from them often fail to capture sufficient species‐specific variation. Several EF1‐α‐based assays exhibited cross‐reactivity with closely related species. For example, the FgramB379fwd/FgramB411rev (Nicolaisen et al. 2009) primer set targeting *F. graminearum* was found to have cross‐reactivity with *F. asiaticum*, leading to potential underestimation of *F. asiaticum* occurrence.

Similarly, functional genes repurposed for species detection (e.g., *Tri5*, *CYP51C*, phosphate permease) demonstrated higher specificity in selected cases but lacked universal applicability (Villafana et al. 2019; Fernández‐Ortuño et al. 2010). Notably, none of the evaluated primers targeting *F. graminearum* achieved the highest primer score of grades (A) under the combined in silico and Primer‐BLAST valuation framework. All published assays exhibited cross‐reactivity, most frequently with *F. asiaticum* and *F. culmorum*. This cross‐reactivity reflects genomic similarity shared among these species and emphasizes the difficulty of enforcing strict species boundaries using short PCR amplicons alone.

In our in silico evaluation, the primer scoring framework considered the total number of primer–template mismatches, but the mismatch position was not directly weighed in the original score calculation. This was because 3′‐end mismatch effects were relevant only to a subset of the primer pairs evaluated, where mismatches occurred within the terminal three bases of the primer 3′ end in either predicted nontarget amplicons or intended target amplicons (Table S2). These cases have different implications for interpretation. Where 3′‐terminal mismatches occurred in predicted nontarget amplicons, the original score may be conservative because such off‐target amplification may be less likely to occur under empirical PCR conditions. Conversely, where the intended target amplicon itself contained a 3′‐terminal mismatch, the original score may overestimate assay reliability because target amplification may be inefficient or inconsistent. Therefore, the original primer scores were retained, and the mismatch position was used as an additional interpretive layer rather than as a recalculated scoring criterion. The affected primer pairs are listed in Table S3.

Taken together, these results expose a critical technological gap and the need to investigate new markers that are universally reliable for species‐level resolution across *Fusarium*. This need stems from the genus's evolutionary complexity and is most acute with recently diverged species complexes, particularly the FGSC, where using traditional short, single‐locus PCR markers has reached a practical “resolution ceiling.” In such cases, reliable discrimination is more likely to come from genome‐informed multilocus marker discovery than from further optimization of individual short amplicons alone, with platforms such as multiplex qPCR, digital PCR (dPCR), or targeted amplicon sequencing serving as practical routes for implementing these discriminatory markers. Overcoming these challenges has been achieved in other taxonomically complex genera, where comparative genomic analyses have been employed to systematically identify regions beyond the canonical barcode loci. For example, genome‐scale comparisons in *Phytophthora* have been used to discover discriminatory loci and inform marker development in the absence of a universally resolving single gene (Cai et al. 2019).

### 5.5 Detection Based on BGCs

In this study, the in silico analysis relied on reported strain metadata for chemotype designation; therefore, the term “chemotype” hereafter relates to a genomic annotation (genotype), and not to a confirmed phenotype based on analytical chemistry validation of the strain.

While legacy assays targeting core genes like *Tri5* are frequently employed as a universal proxy for TRI‐producing potential, our BGC mapping reveals that *Tri5* often persists as a pseudogene or within truncated, nonfunctional clusters in nonproducing genomes. This observation aligns with evolutionary analyses showing that TRI biosynthetic pathways have undergone lineage‐specific diversification, including differential retention, modification, and loss of pathway components across fungi, and that the presence of individual TRI genes does not necessarily indicate a functional biosynthetic capacity (Proctor et al. 2018). In contrast, targeted assays such as the *Tri3* and *Tri12* systems developed by Starkey et al. (2007) demonstrated high specificity by exploiting allelic polymorphism within the FGSC. While these markers are highly effective for their intended lineages, their narrow focus complicates applying them to emerging Type‐A producers (e.g., *F. langsethiae*) (Johns et al. 2022) that fall outside the FGSC.

In this review, the focus was exclusively on DNA‐based PCR approaches for the detection of mycotoxin BGCs. Although RNA‐based (RT‐PCR) approaches targeting TRI and ZEN gene expression have been reported and are biologically informative, offering a window into active biosynthetic activity rather than mere gene (Lysøe et al. 2006), their applicability in food safety contexts is limited. Gene expression is transient and highly condition‐ dependent. Critically, mycotoxin may persist in food matrices long after transcription has ceased. Hence, a negative expression signal, therefore, cannot be interpreted as the absence of contamination, which fundamentally limits the utility of expression‐based approaches for regulatory or surveillance purposes.

### 5.6 qPCR for *Fusarium* Detection and Quantification

qPCR provides a sensitive means to detect and estimate *Fusarium* DNA in wheat and has been widely adopted to approximate fungal biomass and, indirectly, mycotoxin risk. However, quantitative reliability is constrained by extraction efficiency, PCR inhibition, and biological decoupling between fungal DNA and toxin production. Fredlund et al. (2008) systematically evaluated real‐time TaqMan qPCR for the absolute quantification of *F. graminearum* and *F. culmorum* DNA in wheat, demonstrating that quantitative outcomes were strongly influenced by DNA extraction method, PCR inhibition, and standard calibration. Using species‐specific probes originally described by Waalwijk et al. (2004), the authors reported a strong correlation between *F. graminearum* DNA levels and DON contamination (*R* ^2^ = 0.9), whereas *F. culmorum* showed no meaningful association with DON or ZEN. Importantly, the study highlighted that even when PCR efficiency and linearity met accepted performance criteria, differences in extraction yield and matrix‐derived inhibitors resulted in substantial variation in estimated fungal biomass. More broadly, DNA–toxin (metabolite) relationships are further complicated by isolate‐ and host‐specific toxigenic potential, environmental conditions, sampling and subsampling strategies, and the emissions of masked mycotoxins from chemical analyses.

The reporting analysis conducted in the present study further indicates that many published *Fusarium* qPCR assays could improve the sharing of metadata to contextualize these limitations. Underreporting of storage conditions, DNA quality metrics, inhibition testing, and raw Cq data restricts reproducibility. In this regard, the FusaMIQE checklist proposed in this study could provide a framework to distinguish assays that support biologically interpretable quantification from those that offer only qualitative or semiquantitative insights. FusaMIQE is a first step toward compliance with the MIQE guidelines (Bustin et al. 2009), which were recently renewed as MIQE 2.0 (Bustin et al. 2025), and toward developing good practices for the next generation of *Fusarium* diagnostics in agricultural matrices.

### 5.7 Best Practices for Assay Validation and Reporting

A key best practice is to validate assays initially using well‐characterized reference material, ideally including fungal isolates with known genomes and, where relevant, confirmed chemotypes. Such validation allows cycling conditions to be optimized before routine deployment, particularly annealing temperature and extension time. Both of which could strongly influence specificity and amplification efficiency. This is especially important when the assays are intended for use in complex matrices such as wheat matrix or plant tissues, where inhibitors and mixed template background may alter assay behavior. Once implemented, assays should continue to be monitored through periodic inclusion of positive controls and appropriate negative controls, including no template and extraction controls, to detect unexpected amplification events and confirm ongoing assay reliability.

For qPCR‐based workflows, unexpected off‐target amplification may sometimes be identified through melt curve analysis, whereas conventional PCR can often be assessed by product size on gels. Where probe‐based chemistry is feasible, TaqMan assays may offer an additional layer of specificity compared with SYBR Green assays, particularly in settings where cross‐reactivity is a concern. However, no chemistry eliminates assay artifacts, and the choice of platform should be guided by the balance between specificity, cost, throughput, and operational constraints. This is particularly relevant for multiplex assays and chip‐based platforms such as array‐based genotyping systems, where all assays must function under shared cycling conditions and optimization options may be limited. While such systems offer important advantages for throughput, they may also increase the risk of cross‐reactivity or suboptimal amplification if primer sets are not carefully matched to platform constraints.

These considerations reinforce the importance of treating primer selection as only one component of assay quality. A well‐designed diagnostic workflow should also include a detailed method description, transparent reporting of assay conditions, and, where possible, open access to raw data and metadata. This aligns with the purpose of the FusaMIQE framework presented in Table 6, which is not only to evaluate reporting quality retrospectively but also to encourage more robust experimental practice prospectively. In this sense, Table 6 should be viewed as both a reporting checklist and a practical guide for improving reproducibility, comparability, and confidence in *Fusarium* qPCR studies.

Taken together, the best practice for in silico‐guided assay development is to begin with primers that show strong predicted specificity, then verify them empirically under realistic laboratory conditions, using appropriate controls and carefully optimized cycling parameters. This layered approach does not eliminate all risk of false positives, but it substantially reduces the likelihood that off‐target amplification will compromise diagnostic interpretation.

### 5.8 Amplicon‐Based High‐Throughput Sequencing (Metabarcoding) Approaches for *Fusarium* Community Profiling

High‐throughput amplicon sequencing has expanded *Fusarium* surveillance beyond targeted qPCR assays by enabling parallel detection of multiple *Fusarium* species (and co‐occurring fungi) from complex field samples. Rather than targeting one single species, sequencing‐based methods can characterize community composition and reveal co‐infection patterns, which is especially relevant for complex diseases such as FHB. Karlsson et al. (2016) developed *Fusarium‐*specific primers targeting TEF1/EF1α for community profiling, including a new primer pair (FA+7/Ra+6), which was validated on mock community and later field material. This tackled a key early problem, which was low resolution provided by the ITS region for the *Fusarium* genus. Walder et al. (2017) used Pacific Biosciences sequencing of a ∼1600‐bp ribosomal operon amplicon (ITS + D1–D3 LSU) to quantify *Fusarium* down to the species level. This study addressed a different limitation, as short reads may collapse on close taxa, whereas longer amplicons can increase taxonomic signals and help with species calls. However, ribosomal‐marker‐based long‐read amplicons still caused difficulty in closely related *Fusarium* species.

## 6 Recommendations

Based on the reassessment of PCR‐ and qPCR‐based diagnostics presented in this review, several practical recommendations can be made for future *Fusarium* detection and surveillance studies in wheat. With the availability of modern genomic resources, it is now possible to systematically re‐evaluate early PCR markers that continue to perform well by refining primer placements and improving thermodynamic performance and assay specificity. High‐quality, well‐annotated, and taxonomically representative genome assemblies are essential for improving our understanding of this complex genus and for enabling robust, bioinformatically informed diagnostic assay design. At the highest level, whole genome sequencing (WGS) represents the most comprehensive and universally comparable approach for *Fusarium* identification and characterization. Where WGS is not feasible, amplicon‐based sequencing as described by O'Donnell et al. (2015) provides a valuable intermediate solution allowing simultaneous detection of multiple *Fusarium* species within complex samples.

In many applied settings, however, sequencing‐based approaches remain impractical due to cost, infrastructure, or sample constraints. In such cases, PCR‐ and qPCR‐based assays remain essential tools, and their performance can be substantially improved by adopting genome‐informed design principles. In particular, diagnostic assays targeting causal genes directly involved in toxin biosynthesis should be prioritized over proxy markers. For example, *Tri8* represents a more reliable target for differentiating ADON chemotypes than the historically used *Tri12* transporter gene, which does not directly determine toxin production. Similarly, *Tri1*‐based markers may be used to identify NX chemotypes. In this framework, conventional PCR‐ and qPCR‐based approaches may be strengthened by targeted amplicon sequencing, which can provide additional confirmation and support broader taxonomic profiling.

For species‐level detection, no single gene provides universal resolution across all *Fusarium* lineages, particularly within recently diverged species complexes such as the FGSC, FIESC, and FOSC. Therefore, marker selection should be context dependent, and users are encouraged to prioritize assays that have been evaluated against diverse genomic datasets. To facilitate this, the present study highlights a subset of primers with the highest in silico specificity (Table 4), which may serve as a starting point for assay selection. This study also identifies gaps for which reliable species‐specific PCR markers remain unavailable.

In parallel, advances in long‐read sequencing technologies and the expanding availability of *Fusarium* genomic resources offer new opportunities for the development of long‐read amplicon‐based diagnostics that integrate improved taxonomic resolution with functional inference. Emerging approaches, including multiplexed and dPCR platforms, provide practical routes to enhance sensitivity and quantification in *Fusarium* surveillance, particularly when targeting single‐copy genes. Recent work has demonstrated that dPCR assays targeting *Fusarium* species exhibit increased robustness compared with qPCR in oat grain matrices (Natarajan et al. 2025), highlighting the potential of multiplexed, genome‐informed diagnostic frameworks for future *Fusarium* monitoring programs.

## 7 Conclusion

This review systematically re‐evaluated PCR and qPCR‐based molecular approaches for the detection and quantification of mycotoxigenic *Fusarium* species in wheat through the lens of modern genomic resources. By benchmarking existing primers against curated genome panels, this paper has demonstrated that many widely used assays are constrained by taxonomic complexity and historical design assumptions, limiting the specificity and interpretability. A clear distinction between taxonomic detection, chemotype inference, and quantitative estimation is therefore essential to avoid overinterpretation of molecular signals. To support more transparent and reproducible qPCR‐based diagnostics, we developed FusaMIQE, a *Fusarium*‐tailored adaptation of the MIQE guidelines that prioritizes parameters with biological and practical relevance in wheat matrices.

## Author Contributions

**Kavitha Vijeandran**: methodology, software, data curation, formal analysis, writing – original draft, resources, visualization, conceptualization, investigation, writing – review and editing. **Alexey Larionov**: supervision, writing – review and editing, validation, resources, software, funding acquisition, conceptualization. **Carla Cervini**: supervision, writing – review and editing, funding acquisition. **Katrina Campbell**: supervision, writing – review and editing, funding acquisition. **Sean Walkowiak**: writing – review and editing. **Matias Pasquali**: writing – review and editing. **Florence Richard‐Forget**: writing – review and editing. **Neil Brown**: writing – review and editing. **Lindy Joy Rose**: writing – review and editing. **Carol Verheecke‐Vaessen**: methodology, funding acquisition, visualization, conceptualization, supervision, project administration, writing – review and editing.

## Conflicts of Interest

The authors declare no conflicts of interest.

## Supporting information




**Supplementary Material**: crf370612‐sup‐0001‐FigureS1.pdf

**Supplementary Material**: crf370612‐sup‐0002‐TableS1.docx

**Supplementary Material**: crf370612‐sup‐0003‐TableS2.pdf

**Supplementary Material**: crf370612‐sup‐0004‐TableS3.pdf

**Supplementary Material**: crf370612‐sup‐0005‐TableS4.pdf

## Data Availability

The in silico analysis *Fusarium* amplicon finder Python script and data generated in this study are publicly available at: https://github.com/kavithavijeandran/fusarium_amplicon_finder
