# Supplementary material for: Revisiting the PCR‐Based Molecular Approaches for Mycotoxigenic Fusarium Species Detection and Quantification in Wheat
Source: Compr Rev Food Sci Food Saf. 2026 Aug 28;25(5):e70612. doi: 10.1111/1541-4337.70612 (PMC13525088; doi:10.1111/1541-4337.70612)
Supplement: Supplementary file 1 [file CRF3-25-e70612-s001.pdf]

Figure S1.

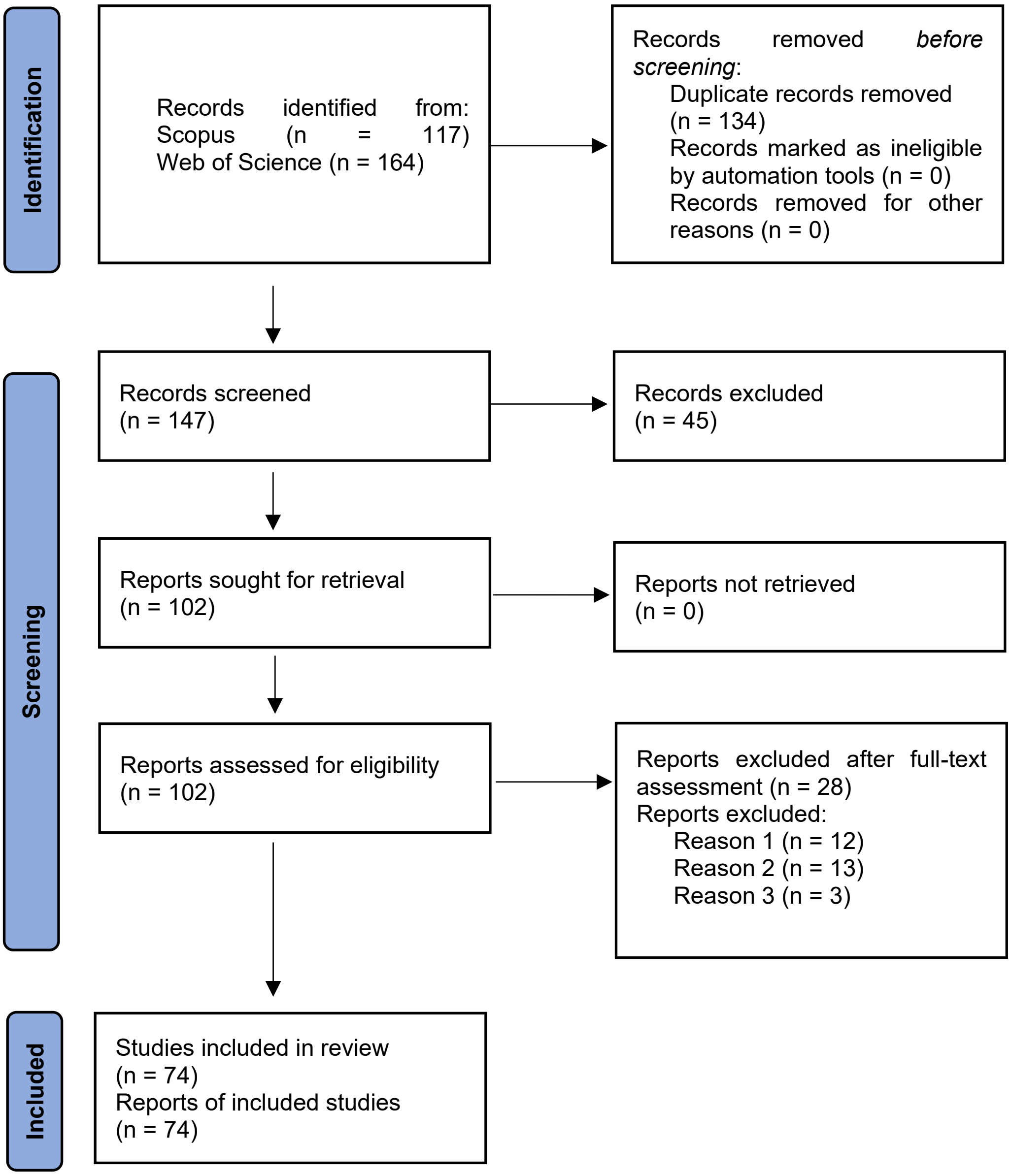

Supplementary Figure S1. PRISMA 2020 flow diagram summarising the literature search and study selection process for PCR/qPCR-based *Fusarium* detection, quantification, and chemotyping assays in wheat. Records were identified from Scopus and Web of Science, screened by title and abstract, assessed by full text, and selected according to the inclusion and exclusion criteria described in the Methods. Full-text exclusion reasons: reason 1: non-wheat host or matrix; reason 2: no new or independently assessable primer set reported; and reason 3: not focused on PCR/qPCR-based *Fusarium* detection, quantification, or chemotyping. Flow diagram adapted from Page et al. (2021).
