# Supplementary material for: Revisiting the PCR‐Based Molecular Approaches for Mycotoxigenic Fusarium Species Detection and Quantification in Wheat
Source: Compr Rev Food Sci Food Saf. 2026 Aug 28;25(5):e70612. doi: 10.1111/1541-4337.70612 (PMC13525088; doi:10.1111/1541-4337.70612)
Supplement: Supplementary file 2 [file CRF3-25-e70612-s002.pdf]

Table S4. Item-level MIQE compliance matrix for published *Fusarium* qPCR assays in wheat-based matrices.

Parameters to report	Rank	Studies															
		1	2	3	4	5	6	7	8	9	10	11	12	13	14	15	16
Study design																	
Experimental and control groups	E	~	✓	✓	✓	✓	✓	✓	✓	✓	✓	✓	✓	✓	✓	✓	✓
Number of biological replicates	E	~	✓	✓	✓	✓	✓	✓	✓	✓	✓	✓	✓	✓	✓	✓	✓
Technical replicates	D	~	-	✓	✓	✓	✓	✓	✓	✓	✓	✓	✓	✓	✓	✓	✓
Sample and nucleic acid quality																	
Sample source and type	E	✓	✓	✓	✓	✓	✓	✓	✓	✓	✓	✓	✓	✓	✓	✓	✓
Storage conditions	E	-	-	-	✓	-	~	✓	-	✓	~	-	~	✓	✓	✓	✓
Extraction method	E	✓	✓	✓	✓	✓	✓	✓	✓	✓	✓	✓	✓	✓	✓	✓	✓
DNA quality metrics	E	~	✓	✓	~	-	~	✓	-	~	-	-	-	✓	✓	✓	~
Inhibition check	D	-	✓	✓	✓	~	✓	✓	✓	~	✓	✓	~	✓	-	✓	~
Primer and target information																	
Target gene / cluster	E	✓	✓	✓	✓	✓	✓	✓	✓	✓	✓	✓	✓	✓	✓	✓	✓
Amplicon length and position	E	✓	~	✓	✓	✓	~	✓	✓	✓	~	✓	✓	✓	✓	✓	✓
Primer sequences and source	E	✓	✓	✓	✓	✓	✓	✓	✓	✓	✓	✓	✓	✓	✓	✓	✓
Empirical validation	E	✓	✓	✓	✓	✓	✓	✓	✓	✓	✓	✓	✓	✓	✓	✓	✓
Chemotype coverage*	D	-	-	-	✓	-	-	-	-	-	✓	-	✓	-	-	-	-
In-silico validation	D	-	✓	✓	-	-	✓	✓	✓	✓	✓	✓	✓	✓	-	✓	✓
qPCR protocol																	
Reaction conditions	E	✓	✓	✓	✓	✓	✓	✓	✓	✓	✓	✓	✓	✓	✓	✓	✓
Thermocycler model & cycling profile	E	✓	✓	✓	✓	✓	✓	✓	✓	✓	✓	✓	✓	✓	✓	✓	✓
Calibration curve and efficiency	E	~	~	~	~	~	~	✓	✓	✓	✓	✓	✓	✓	-	✓	✓
Limit of detection (LOD)	D	✓	✓	✓	✓	✓	✓	✓	✓	✓	✓	✓	✓	✓	✓	✓	✓
Data analysis																	
Negative & positive controls	E	✓	✓	✓	✓	✓	✓	✓	✓	✓	✓	✓	✓	✓	-	✓	~
Cq/Ct determination method	E	~	✓	✓	✓	✓	~	✓	✓	✓	✓	✓	✓	✓	✓	✓	✓
Quantification model	E	✓	✓	✓	✓	✓	✓	✓	✓	✓	✓	✓	✓	✓	✓	✓	✓
Biological vs. technical variation	D	✓	✓	✓	✓	✓	✓	✓	~	~	✓	✓	~	✓	✓	✓	✓
Raw data availability	D	-	-	-	-	-	-	-	-	-	-	-	-	-	-	✓	✓
Reporting and reproducibility																	
Interpretation context	E	✓	✓	✓	✓	✓	✓	✓	✓	✓	✓	✓	✓	✓	✓	✓	✓
Chemotype inference limits*	E	~	~	~	~	~	~	✓	✓	-	✓	✓	✓	✓	✓	✓	✓
Primer metadata availability	D	✓	✓	✓	✓	✓	✓	✓	✓	✓	✓	✓	✓	✓	✓	✓	✓

Rank: E indicates essential FusaMIQE parameters, while D indicates desirable parameters. Symbol definitions: "✓" indicates that the parameter was explicitly reported, "~" indicates the parameter are partially reported and "-" indicates the parameter was not reported. References (Studies 1-16): 1. Schnerr et al. (2001), 2. Waalwijk et al. (2004), 3. Leišová et al. (2006), 4. Burlakoti et al. (2007), 5. Yin et al. (2009), 6. Moradi et al. (2010), 7. Stakheev et al. (2011), 8. Horevaj et al. (2011), 9. Kulik (2011), 10. Hamada et al. (2012), 11. Vegi and Wolf-Hall (2013), 12. Kulik et al. (2015), 13. Kumar et al. (2015), 14. Morcia et al. (2020), 15. Thomas et al. (2022), and 16. Wang et al. (2023).
