# Supplementary material for: Revisiting the PCR‐Based Molecular Approaches for Mycotoxigenic Fusarium Species Detection and Quantification in Wheat
Source: Compr Rev Food Sci Food Saf. 2026 Aug 28;25(5):e70612. doi: 10.1111/1541-4337.70612 (PMC13525088; doi:10.1111/1541-4337.70612)
Supplement: Supplementary file 3 [file CRF3-25-e70612-s003.pdf]

Table S2. Genome assemblies used for *in silico* primer evaluation of *Fusarium* species.

Species name	Accession	Assembly	Strain Name	Size (Mb)	Assembly Level	Chemotype/ Mycotoxin Profile	Culture Collection	Tier	BUSCO		QUAST
									C	S	N50
F. acuminatum	GCA_038181435.1	ASM3818143v1	A1	46.7	Complete Genome	No record	No record	C	99.4	99.2	9989830
F. acuminatum	GCA_027247665.1	ASM2724766v1	CHS3	44	Contig	No record	No record	E	99.6	99.3	5020484
F. asiaticum	GCA_025258505.1	ASM2525850v1	KCTC 16664	38.3	Complete Genome	NIV	Korean collection for type cultures	C	99.5	99.4	9437819
F. asiaticum	GCA_050916195.1	ASM5091619v1	180197	38.4	Chromosome	3-ADON	No record	C	99.6	99.5	9351427
F. avenaceum	GCA_025948275.1	ASM2594827v1	WV21P1A	42	Complete Genome	No record	USDA ARS	C	99.6	99.4	9183391
F. cerealis	GCA_019055205.1	ASM1905520v1	S18/34	36.5	Contig	No record	No record	E	99.5	99.4	490630
F. culmorum	GCA_900074845.1	FcUK99v1.2	UK99	41.9	Chromosome	3-ADON	Rothamsted Research collections,	C	98.6	98.6	9025752
F. culmorum	GCA_016952355.1	ASM1695235v1	Class2-1B	37.5	Complete Genome	No record	Chinese Academy of Forestry	E	99.5	99.5	9630554
F. equiseti	GCA_003313175.1	ASM331317v1	D25-1	40.8	Contig	TRI & ZEN	No record	C	99.4	99.3	8914601
F. equiseti	GCA_038501615.1	ASM3850161v1	HG18	38.5	Contig	No record	China center for type culture collection (CCTCC)	E	99.4	99.4	3843907
F. graminearum	GCA_023242275.1	ASM2324227v1	NRRL 29169	36.8	Chromosome	15-ADON, ZEN	Westerdijk CBS 110266	C	99.4	99.3	8840934
F. graminearum	GCA_900073075.1	CML3066.v2	CML3066	36.9	Complete Genome	15-ADON	No record	C	99.5	99.4	8641347
F. graminearum	GCF_000240135.3	ASM24013v3	PH-1; NRRL 31084	36.5	Chromosome	15-ADON	Westerdijk CBS 123657	C	98.7	98.6	6676775
F. graminearum	GCA_019343145.1	ASM1934314v1	FG-12	35.9	Chromosome	No record	No record	E	99.6	99.5	9045406

F. graminearum	GCA_037213105.1	ASM3721310v1	KSU23522	37.1	Complete Genome	No record	No record	E	99.5	99.4	9010877
F. graminearum	GCA_037255895.1	ASM3725589v1	KSU23473	36.8	Complete Genome	No record	No record	E	99.5	99.4	8894400
F. langsethiae	GCA_018104275.1	ASM1810427v1	Fe2391	59.6	Contig	T-2/HT-2	No record	C	99.5	99.1	6178397
F. oxysporum	GCA_030345115.2	ASM3034511v2	Fo5176	69.6	Complete Genome	No record	No record	E	99.6	97.8	4523040
F. oxysporum	GCF_013085055.1	ASM1308505v1	Fo47	50.4	Complete Genome	No record	No record	E	99.5	99.1	4366112
F. poae	GCA_046056315.1	ASM4605631v1	Fp133	44.9	Complete Genome	NIV	DAOMC (Canadian collection)	C	99.5	99.4	6113431
F. poae	GCF_019609905.1	ASM1960990v1	DAOMC 252244	43.9	Chromosome	NIV	DAOMC (Canadian collection)	C	99.5	99.4	5140998
F. proliferatum	GCA_036288945.1	ASM3628894v1	NRRL62905	43.6	Complete Genome	FB1, BEA, ENNs, MON, FUP	ARS Culture Collection	C	99.5	99.3	4957983
F. proliferatum	GCA_047651765.1	ASM4765176v1	ZO-L2-4	43.5	Chromosome	No record	No record	E	99.5	99.4	4293844
F. proliferatum	GCF_900067095.1	ET1v1	ET1	45.2	Scaffold	Fusarin & fujikurin	No record	E	99.6	99.2	3311891
F. pseudograminearum	GCF_000303195.2	FP7	CS3096	36.9	Chromosome	3ADON	CSIRO	C	99.4	99.4	4351731
F. pseudograminearum	GCA_016952305.1	ASM1695230v1	Class2-1C	37.8	Complete Genome	No record	No record	E	99.6	99.5	9153420
F. sambucinum	GCA_050947815.1	OSU_Fsam_2.1	potato_lamoka	38.6	Complete Genome	No record	Oregon State University'	E	99.6	99.5	9651250
F. sporotrichioides	GCA_019054645.1	ASM1905464v1	S17/16	38	Scaffold	No record	No record	E	99.4	99.4	9051733
F. tricinctum	GCA_020744515.1	Fustr1	MPI-SDFR-AT-0068	43.7	Contig	No record	No record	E	99.4	99.2	5209653
F. verticillioides	GCA_031360185.1	ASM3136018v1	ITEM 10027	44.7	Complete Genome	Fumonisin (FB1)	CNR, Bari, Italy (ITEM collection)	C	99.5	99.3	4236349

F. verticillioides	GCF_000149555.1	ASM14955v1	7600	41.8	Chromosome	Fumonisin (FB1)	ARS Culture Collection	C	99.4	99.3	3513144

Tier indicates the genome selection category used in this study: C (core) represents high-quality genome assemblies, while E (expended) represents additional assembles included to broaden species and chemotype coverage. BUSCO refers to Benchmarking Universal Single-Copy Orthologs metrics, where C indicates the percentage of complete BUSCO genes and S indicates the percentage of those that are present as single-copy orthologs. QUAST N50 represents the contig or scaffold length (in base pairs) at which 50% of the total assembly length is contained in contigs or scaffolds of that length or longer.
