# Supplementary material for: Revisiting the PCR‐Based Molecular Approaches for Mycotoxigenic Fusarium Species Detection and Quantification in Wheat
Source: Compr Rev Food Sci Food Saf. 2026 Aug 28;25(5):e70612. doi: 10.1111/1541-4337.70612 (PMC13525088; doi:10.1111/1541-4337.70612)
Supplement: Supplementary file 4 [file CRF3-25-e70612-s005.pdf]

Table S3. Primer pairs where mismatches occur within the first three bases of the 3' end.

| Target species | Primer pair | Original score | 3' Mismatch Category | References |
|---|---|---|---|---|
| *F. graminearum* | FgramB379fwd/FgramB411rev | **D** | Off-target | (Nicolaisen et al., 2009) |
| | Fgtubf/Fgtubr | **D** | Off-target | (Sanoubar et al., 2015) |
| | UBC85F/UBC85R | **E** | Off-target | (Schilling et al., 1996) |
| | COB1/COB2* | **D** | Off-target | (Kulik et al., 2015) |
| *F. culmorum* | FculC561_fwd/FculC614_rev* | **D** | Off-target | (Nicolaisen et al., 2009) |
| *F. avenaceum* | FAF1/FAR | **D** | Target | (Mishra et al., 2003) |
| | Fave574_fwd/Fave627_rev* | **D** | Target | (Nicolaisen et al., 2009) |
| *F. sporotrichioides* | FspoA18_fwd/FspoA85_rev* | **D** | Off-target | (Nicolaisen et al., 2009) |
| | Tox5-1/Tox5-sporo_R2 | **B** | Target | (Niessen and Vogel 2010) |
| *F. pseudograminearum* | FPG_F/FPG_R | **E** | Off-target | (Williams et al., 2002) |
| *F. proliferatum* | Fu-4F/Fprol-R | **C** | Off-target | (Deng et al., 2024) |
| *F. langsethiae* | FlangA29_fwd/FlangA95_rev* | **D** | Off-target | (Nicolaisen et al., 2009) |
| *F. cerealis* | Fcerf1/Fcerr1 | **C** | Off-target | (Fernández-Ortuño et al., 2010) |

The 3' mismatch category was interpreted as follows: "Off-target" indicates that a predicted non-target amplification contains a mismatch within the terminal three bases of the primer 3' end and was therefore interpreted as lower-confidence in predicted off-target amplification. "Target" indicates that the predicted amplification of the intended target species contains a mismatch within the terminal three bases of the primer 3' end; in these cases, the original primer score may overestimate primer reliability.
