# Supplementary material for: Revisiting the PCR‐Based Molecular Approaches for Mycotoxigenic Fusarium Species Detection and Quantification in Wheat
Source: Compr Rev Food Sci Food Saf. 2026 Aug 28;25(5):e70612. doi: 10.1111/1541-4337.70612 (PMC13525088; doi:10.1111/1541-4337.70612)
Supplement: Supplementary file 5 [file CRF3-25-e70612-s004.docx]

Table S1. Legacy and advanced PCR-based techniques used for molecular detection and characterisation of *Fusarium*

| Technique | Principle | Main advantages | Main limitations | Examples |
| --- | --- | --- | --- | --- |
| Random amplified polymorphic DNA PCR | Uses short arbitrary primers to amplify multiple random genomic regions without requiring prior sequence information. Products are separated by gel electrophoresis to generate target-specific banding profiles. | Simple, inexpensive and useful for preliminary differentiation of isolates. | Limited reproducibility, banding patterns may be difficult to interpret | (Voigt et al. 1995) |
| Species-specific endpoint PCR | Uses primers designed to amplify a taxon-specific genomic region, with products usually detected by gel electrophoresis. | Simple, inexpensive and widely accessible; enables targeted presence-or-absence detection. | Primarily qualitative; dependent on primer specificity | Primer assays evaluated in this review (Tables 2.4). |
| Nested PCR | Uses two successive amplification reactions, with an outer primer pair followed by an internal primer pair targeting the first amplicon. | Improved analytical sensitivity and specificity for low-abundance targets. | Increased handling time and risk of carry-over contamination; generally, not quantitative. | (Klemsdal and Elen 2006) |
| Multiplex PCR | Uses multiple primer pairs in a single reaction to amplify several specific targets simultaneously. | Reduces time, reagent use and sample consumption; enables simultaneous screening of multiple targets. | Primer competition can reduce amplification efficiency and sensitivity among targets; complex assay optimisation as additional primer pairs are included. | (Mishra et al. 2003; Neera and Murali 2021; Deng et al. 2024) |
| Quantitative real-time PCR (qPCR) | Monitors amplification in real time using intercalating dyes or sequence-specific fluorescent probes. Target abundance is estimated using amplification thresholds and, typically, a calibration curve. | Sensitive, rapid and quantitative; closed-tube format reduces contamination risk; suitable for high-throughput analysis. | Requires specialised equipment and appropriate standards; affected by inhibitors, amplification efficiency and target copy-number variation. | qPCR studies evaluated in this review (Supplementary Table S4) |
| Digital PCR (dPCR) | Partitions the sample into thousands of individual reactions and determines target copy number from the proportion of positive and negative partitions using Poisson statistics. | Enables absolute quantification without standard curve; provides improved precision and inhibitor tolerance; useful for low-abundance targets. | High equipment and consumable costs; limited dynamic range if partitions become saturated; accuracy depends on target copy-number stability and assay design. | (Natarajan et al. 2025) |
| Multilocus amplicon sequencing | Simultaneously or sequentially amplifies multiple informative loci, such as TEF1α, RPB1, RPB2 or other genomic regions, followed by sequencing and combined taxonomic analysis. | Improves species-level resolution and confidence compared with a single marker; can distinguish closely related taxa. | Increased assay complexity, cost and bioinformatic requirements; incomplete reference data may limit interpretation. | (O’Donnell et al. 2015) |
| Targeted long-read amplicon sequencing | Amplifies long genomic regions spanning multiple variable sites or linked loci and sequences the complete amplicon using long-read platforms. | Preserves linkage across informative variants; can improve species resolution and support mixed-community analysis. | Higher cost; requires specialised library preparation and bioinformatics. | (Karlsson et al. 2016; Walder et al. 2017) |
